# Synthesis and antioxidant, antimicrobial, and antiviral activity of some pyrazole-based heterocycles using a 2(3*H*)-furanone derivative

**DOI:** 10.1007/s13738-023-02814-w

**Published:** 2023-06-03

**Authors:** Youssef M. Youssef, Mohammad E. Azab, Galal A. Elsayed, Amira A. El-Sayed, Aya I. Hassaballah, Mounir M. El-Safty, Reem A. Soliman, Eman A. E. El-Helw

**Affiliations:** 1 Chemistry Department, Faculty of Science, Ain Shams University, Cairo, 11566 Egypt; 2 Department of Evaluation of Inactivated Viral Poultry Vaccines, Department of Quality Control of SPF Eggs, Central Laboratory for Evaluation of Veterinary Biologics, Agriculture Research Center (ARC), Giza, Egypt; 3 Department of Evaluation of Inactivated Viral Poultry Vaccines, Department of Quality Control of SPF Eggs, Central Laboratory for Evaluation of Veterinary Biologics, Agriculture Research Center (ARC), Giza, Egypt

**Keywords:** 5-Chloropyrazole, Antiviral, Pyrrolone, Pyridazinone, Antioxidant

## Abstract

Some pyrazole-based heterocycles such as pyrrolone, pyridazinone, and imidazole derivatives were synthesized utilizing the pyrazolyl-2(3*H*)-furanone derivative **3**, which was obtained in a good yield via Perkin condensation of 5-chloro-4-formyl-3-methyl-1-phenylpyrazole with 3-(4-methylbenzoyl)propionic acid in the presence of cyclo-dehydrating agent (sodium acetate and acetic anhydride). Also, the acid hydrazide obtained was reacted with some carbonyl reagents such as acetic anhydride, benzoyl chloride, 4-chlorobenzaldehyde, and 1,3-diphenyl-4-formylpyrazole, aiming to achieve new pyrrolone derivatives. The antioxidant, antimicrobial, and antiviral activity screening of some synthesized compounds demonstrated that some of them offered strong potencies. An experimental trial was undertaken to explore the effect of different substances and applied against avian influenza HPAI-H5N1 (AIV) to evaluate the antiviral replication in specific pathogen-free chicken embryos. All the compounds were screened for their antimicrobial activities, and most of tested compounds showed potent inhibition growth activity toward *Haemophilus* (Gram-negative bacteria), *Staphylococcus aureus* (Gram-positive bacteria), and *Candida albicans* fungus. Upon antimicrobial screening, it was observed that the majority of the compounds were found to be active against *Staphylococcus aureus*, *Haemophilus*, and *Candida albicans* as compared to standard drugs. This experiment shows the potential usage of these compounds as antiviral agents and can be considered as a viable means to control the economically important avian influenza of poultry. These compounds can thus be recommended for their antiviral, antibacterial, and antifungal property and can very well be used as immunostimulants.

**Supplementary Information:**

The online version contains supplementary material available at 10.1007/s13738-023-02814-w.

## Introduction

Pyrazoles represent important classes of the heterocyclic systems because of their synthetic and pharmacological properties including antiviral, anticancer, antioxidant, insecticidal, and antimicrobial activities [1–9]. The 2(3*H*)-furanones, which are widely recognized as essential components of natural products, were easily converted into a variety of nitrogen heterocycles [10–14]. Also, pyrrolones offered a broad range of pharmacological properties [15]. The pyrrole ring is widely spread and incorporated into many naturally occurring compounds such as chlorophyll, heme, vitamin B_12_, and the bile pigments. *Pyrrolnitrin* and *pyoluteorin* are naturally occurring pyrroles which have antibiotic activity, and the ester methyl 4-methylpyrrole-2-carboxylate is an insect pheromone [16, 17].

**Fig. 1:**
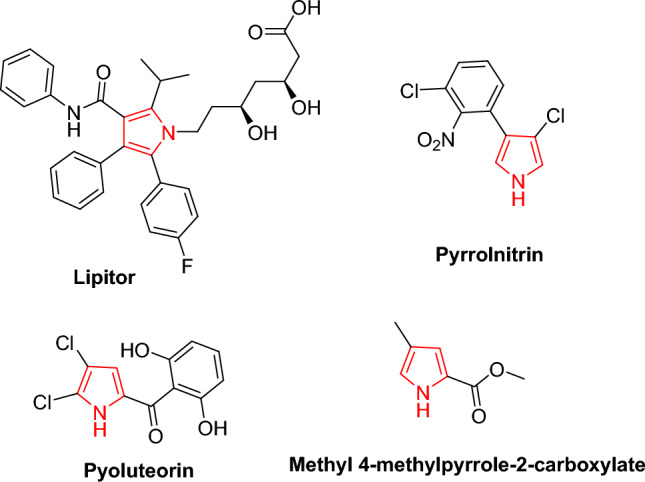
Some potent pyrrole-based pharmaceuticals

Also, the highly successful cholesterol lowering drug *Lipitor* is a poly-substituted pyrrole derivative [18, 19]. Figure 1 depicts some pyrrole-based drugs. Otherwise, high levels of free radicals and reactive oxygen species can harm cell membranes and tissues, as likewise proteins, lipids, enzymes, and DNA. To forestall free radical damage, the cell antioxidants should be neutralized. Furthermore, free radicals, reactive oxygen species, and oxidative stress all contribute to brain maturation and age-related neurodegenerative diseases such as Alzheimer’s [20]. They have been linked to several chronic diseases, including atherosclerosis, coronary heart disease, and cancer [21]. There are claims that antioxidants protect the brain from Alzheimer's disease.

In turn, poultry is the largest livestock sector among the domestic animal stock in the world [22], accounting for more than 30% of all animal protein [23]. The discovery of new substances with proven antimicrobial and antiviral activity is the current study goal of various researchers. Usage of synthetic products has grown considerably in the past few years due to capability of going through previous chemical modifications in to enhance its biological activity. Due to the large raise of pathogenic microorganisms’ resistance to multiple drugs, there is a concern on finding new therapeutic alternatives. Many compounds have been used for many decades in health care for treatment of different microbial infections. Some antiviral compounds inhibit the AI virus’s entry into host cell [24].

Influenza A viruses are divided into subtypes based on the antigenic relationships of the surface glycoproteins, hemagglutinins (HA, H1–H17), and neuraminidases (NA, N1–N10) [25]. Among these, some strains of the H5 and H7 subtypes are the primary cause of highly pathogenic avian influenza (HPAI), which results in systemic infections with high mortality rates in gallinaceous birds. Studies have reported that the HPAI viruses can infect a wide range of domestic poultry under natural and experimental conditions, and susceptibility to HPAI can vary greatly among bird species [26].

The rapidly changing influenza virus has remained a consistent threat to the well-being of a variety of species on the planet. Influenza virus’ high mutation rate has allowed the virus to evolve rapidly and continuously, as well as generate new strains that are resistant to the current commercially available antivirals. Thus, the increased resistance has compelled the scientific community to explore alternative compounds that have antiviral effects against influenza virus [27]. Vaccination remains one of the central public health interventions to combat seasonal influenza. However, vaccine development lead times of at least 6 months limit their applicability during outbreaks of novel influenza viruses. Antiviral compounds are useful interventions against novel influenza viruses, and they can reduce disease burden caused by seasonal strains [28].

*Staphylococcus aureus* is a major opportunistic pathogen in humans and one of the most important pathogenic *Staphylococcus* species in veterinary medicine. *S. aureus* is dangerous because of its deleterious effects on animal health and its potential for transmission from animals to humans and vice versa. It thus has a huge impact on animal health and welfare and causes major economic losses in livestock production. Increasing attention is therefore being paid to both livestock and companion animals in terms of this pathogen.

*Staphylococcus aureus* is a Gram-positive spherically shaped bacterium, a member of the Bacillota, and is a usual member of the microbiota of the body, frequently found in the upper respiratory tract and on the skin. It is often positive for catalase and nitrate reduction and is a facultative anaerobe that can grow without the need for oxygen. Although *S. aureus* usually acts as a commensal of the human microbiota, it can also become an opportunistic pathogen, being a common cause of skin infections including abscesses, respiratory infections such as sinusitis and food poisoning [29]. *Haemophilus* species are morphologically variable Gram-negative bacilli, ranging from short rods to long filaments. They are facultative anaerobes and are typically oxidase positive. They depend on beta-nicotinamide adenine dinucleotide (NAD) (V factor) and/or haemin (X factor) for growth. Heated blood agar (chocolate agar) is required for growth of NAD-dependent strains. Haemin-dependent strains grow on blood agar but do not grow on MacConkey agar. The *Haemophilus* species inhabit the mucosal epithelium of the upper respiratory and lower genital tract. They cause suppurative infections due to the release of pro-inflammatory cytokines, released from macrophages. This includes serofibrinous to fibrinosuppurative infections of the lungs, body cavities, and joints. The colonization of meningeal vessels causes thrombotic vasculitis leading to encephalitis and meningitis.

Motivated by these promising aspects and in continuation to our research interest [30–40], our rational design was based on a structural diversification through conserving the pyrazole scaffold with different side chain or heterocyclic moieties, to attain an active antioxidant agent with an improved activity. Therefore, a 2(3*H*)-furanone-bearing 5-chloropyrazole scaffold **3** has been synthesized and submitted to react with various nitrogen nucleophiles aiming to obtain some new pyrazole-based heterocycles such as pyrrolone, pyridazinone, and imidazole derivatives to investigate their antioxidant and antiviral activity.

## Results and discussion

### Chemistry

**Scheme 1.**
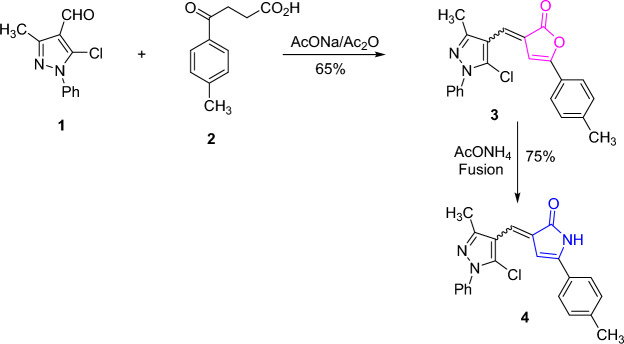
Synthesis of furanone and pyrrolone derivatives **3** and **4**

The key starting material, 5-chloropyrazolyl-2(3*H*)-furanone derivative **3**, was synthesized in a good yield via Perkin condensation of 5-chloro-4-formyl-3-methyl-1-phenylpyrazole **(1)** [41] with 3-(4-methylbenzoyl)propionic acid **(2)** [42] in the presence of anhydrous sodium acetate and acetic anhydride (as cyclo-dehydrating agent) (Scheme 1). The IR spectrum of **3** provided the absorption band for lactone carbonyl group at ν 1761 cm^−1^. Also, its ^1^H NMR spectrum displayed the characteristic singlet signals for methyl protons linked to pyrazole at δ 2.41 ppm and methyl protons of tolyl group at δ 2.36 ppm. The proclivity of furanone derivative **3** was investigated against some mono- and bidentate nitrogen nucleophiles. Initially, fusion of furanone **3** with ammonium acetate in a sand bath afforded the corresponding pyrrolone derivative **4** (cf. Scheme 1). The IR spectrum of pyrrolone **4** lacked to the lactone absorption and displayed the amide carbonyl absorption at ν 1689 cm^−1^, as well as the NH absorption at ν 3179 cm^−1^. Also, its ^1^H NMR spectrum showed an exchangeable singlet signal for NH proton at δ 10.50 ppm.

**Scheme 2.**
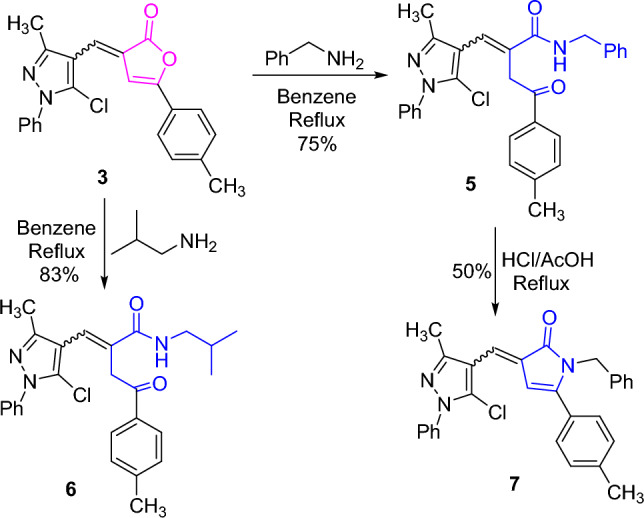
Reactions of furanone derivative **3** with benzylamine and isobutylamine

On the other hand, interaction of furanone derivative **3** with benzylamine and isobutylamine furnished the corresponding amides **5** and **6**, respectively. The *N*-benzylamide derivative **5** was successfully transformed into its *N*-benzylpyrrolone derivative **7** upon heating with a mixture of hydrochloric and acetic acids (Scheme 2). The lactone absorptions were absent from the IR spectra of **5** and** 6**. Further, their ^1^H NMR spectra disclosed exchangeable singlet signals for NH protons at δ 6.77 and 6.62 ppm, respectively. The chemical structure **7** was ascertained by its ^1^H NMR spectrum which lacked the exchangeable singlet signal of NH proton (cf. “Experimental”).

**Scheme 3.**
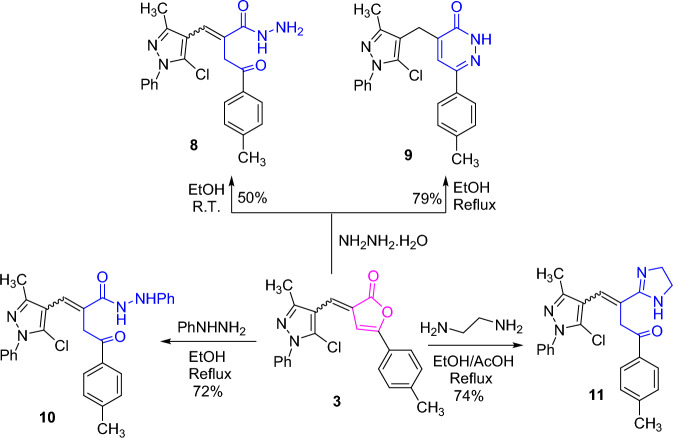
Behavior of furanone derivative **3** toward some bidentate nucleophiles

Notably, furanone derivative **3** was subjected to reactions with some bidentate nucleophiles such as hydrazine, phenylhydrazine, and 1,2-diaminoethane. Initially, stirring an ethanolic solution of **3** with hydrazine hydrate at room temperature furnished a good yield of the acid hydrazide **8**. Meanwhile, under refluxing condition, the pyridazinone derivative **9** was provided. Next, treating an ethanolic solution of furanone **3** with phenylhydrazine achieved the *N*-phenylhydrazide derivative **10**. The IR spectrum of **10** displayed the NH and C=O absorptions. Condensation of furanone **3** with 1,2-diaminoethane under refluxing condition with traces of acetic acid yielded the imidazole derivative **11** (cf. Scheme 3).

**Scheme 4.**
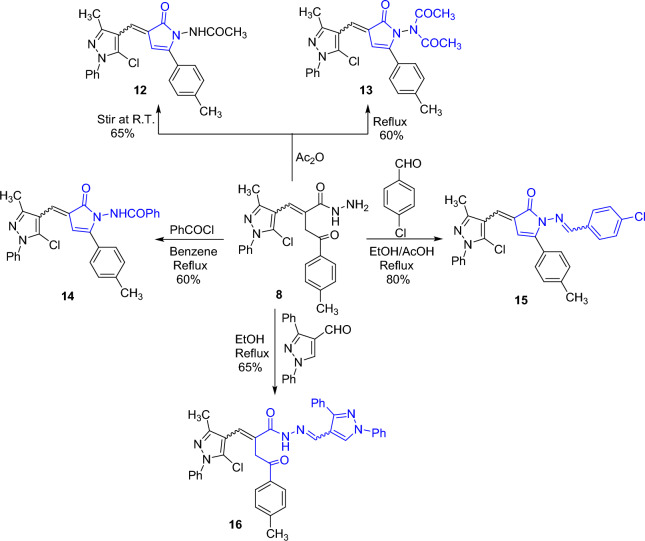
Synthesis of pyrrolone derivatives **12–15** and hydrazone derivative **16**

**Scheme 5.**
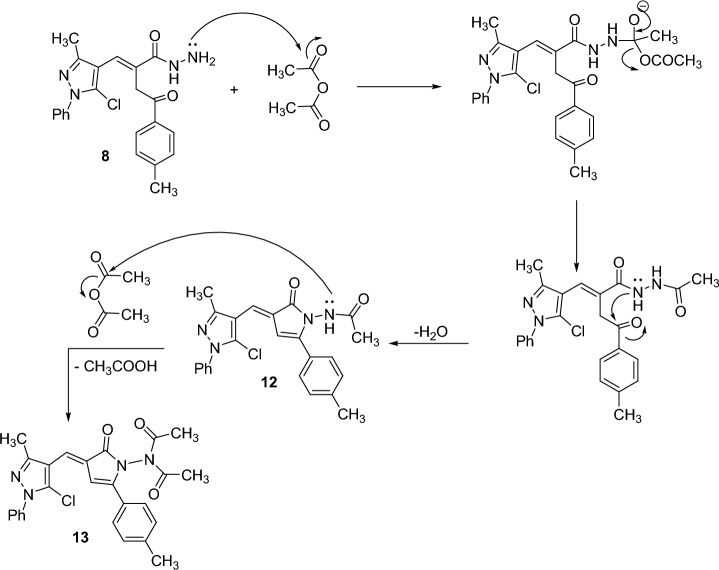
Plausible mechanistic pathways for compounds **12** and **13**

The reactions of the obtained acid hydrazide **8** with some carbonyl reagents such as acetic anhydride, benzoyl chloride, 4-chlorobenzaldehyde, and 1,3-diphenyl-4-formylpyrazole were investigated. Thus, acylation of hydrazide **8** with acetic anhydride at an ambient temperature acquired the *N*-monoacetylpyrrolone derivative **12**, while under refluxing condition, the *N,N*-diacetylpyrrolone derivative **13** was afforded. The ^1^H NMR spectrum of **13** lacked the NH signal. The formation of compounds **12** and **13** is displayed in Scheme 5. Refluxing **8** with benzoyl chloride in benzene offered the *N*-benzoylpyrrolone derivative **14**. Condensation of **9** with 4-chlorobenzaldehyde in the presence of acetic acid gave pyrrolone derivative **15**. Finally, condensation of **9** with 1,3-diphenyl-4-formylpyrazole in boiling ethanol furnished the hydrazone derivative **16** (Scheme 4).

### Antioxidant activity assessment

**Fig. 2:**
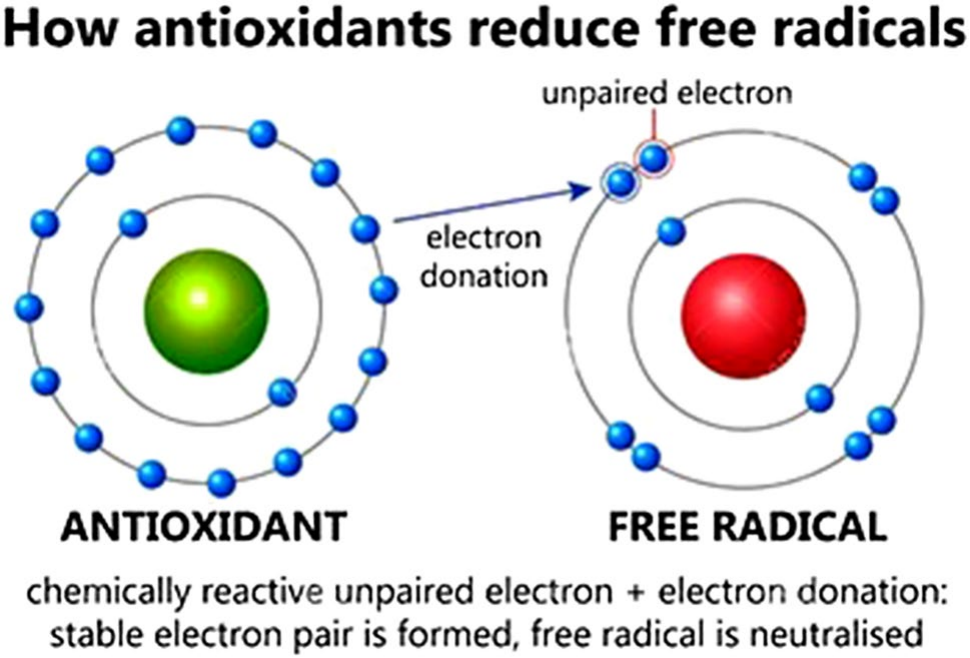
How antioxidant reduce free radicals

Antioxidants are compounds which can inhibit or prevent the oxidation of materials that can be oxidized by scavenging free radicals and help in diminishing oxidative stress. The antioxidant activity is more intriguing since the high levels of free radicals can harm cell membranes and tissues, as likewise proteins, lipids, enzymes, and DNA. To forestall free radical damage, cell antioxidants should be neutralized (cf. Fig. 2). The synthesized compounds were tested for antioxidant activity according to phosphomolybdenum method using ascorbic acid as standard [43, 48]. The antioxidant activity of the sample was expressed as the number of ascorbic acid equivalents (AAE). The results are depicted in Table 1. These values ranged from 121.39 to 374.0 mg AAE/g sample.

**Table 1:** Total antioxidant capacity (TAC) of the tested compounds

Compound	Total antioxidant capacity (mg AAE/g dry compound)^1,2^
3	156.33 ± 4.04
4	*278.56 ± 4.61*
5	*221.14 ± 4.63*
6	*248.67 ± 4.11*
8	145.0 ± 1.73
9	**374.0 ± 2.08**
10	**336.0 ± 3.46**
11	*280.0 ± 2.0*
12	163.0 ± 1.75
13	190.0 ± 1.67
14	*261.0 ± 2.48*
15	*262.27 ± 2.51*
16	*205.82 ± 2.45*

^1^Results are (means ± S.D.) (*n* = 3)

^2^*AAE* ascorbic acid equivalent

**Fig. 3:**
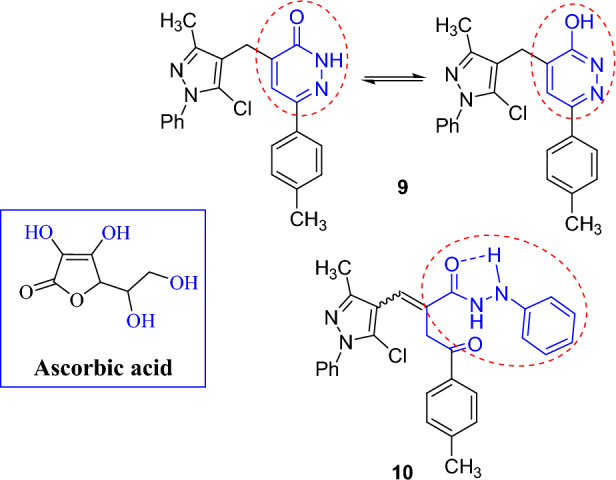
SAR of the most potent compounds

The following points have been highlighted: (1) All compounds were discovered to be effective. (2) Two compounds **9** and **10** demonstrated the highest potent levels of activity. The higher potency of pyridazinone derivative **9** could be attributable for the existence of tautomeric structures and the phenolic hydroxyl group. In turn, the more potency of *N*-phenylhydrazide derivative **10** could be attributed to the additional phenyl group, hydrogen bonding, and extended conjugation linked with the hydrazide skeleton (cf. Fig. 3) [4]. (3) Seven compounds **4–6, 11,** and** 14**–**16** showed exceptional activity. (4) Moderate antioxidant activity was shown by compounds **3, 8, 12,** and **13**. This is coherent with the literature that reported good antioxidant activities by the pyridazine and *N*-phenylhydrazide derivatives [44, 45]. For example, 8-amino-5-chloro-7-phenylpyrido[3,4,d]pyridazine-1,4-(2*H*,3*H*)-dione detected superoxide, peroxynitrite, and hydrogen peroxide in cell free systems as well as in isolated mitochondria [46]. 5-Substituted pyrrolo[1,2-b]pyridazines provided profound inhibition of lipid peroxidation in vitro [44].

### Antiviral activity

Influenza remains a significant threat to global public health. One of the important factors behind this threat is the unpredictability of influenza viruses. Even with global networks monitoring the circulation of viruses in humans and animals, it is not possible to predict with accuracy the emergence of new viruses that could have the potential to cause outbreaks or even pandemics. It is in a rapidly evolving situation such as an epidemic or pandemic where antivirals play a particularly important role.

### The different inhibition concentration fifty in embryonated chicken SPF eggs and their embryonic toxicity

In each case of the tested substance, the toxicity assays in embryonated chicken specific pathogen-free (SPF) eggs indicated that at a concentration (CC_50_) ranging from 300 to 500 µl/ egg (Table 2). The recorded therapeutic index ranged from 50 to 96.77.

**Table 2:** The different inhibition concentration fifty in embryonated chicken SPF eggs and their embryonic toxicity

Substance	CC_50_	IC_50_	TI
*AIV*			
3	300 >	3.1 ≤	96.77
4	500 >	6 ≤	83.3
5	300 >	3.1 ≤	96.77
6	500 >	6 ≤	83.3
8	500 >	9 ≤	55.5
9	400 >	8 ≤	50
10	500 >	9 ≤	55.5
11	500 >	6 ≤	83.3
13	500 >	7 ≤	71.42
14	500 >	9 ≤	55.5
15	500 >	9 ≤	55.5
16	500 >	6 ≤	83.3

Twelve substances showed antiviral activity. Embryonated eggs inoculated with a mixture of AIV with each substance separately affect the virus replication, and the allantoic fluid from each group of eggs receiving varying serial dilutions of the mixtures showed that reduction of viral titer and that lead to lose of its ability for hemagglutination in the treated group in comparison with control group as presented in Table 3.

**Table 3:** Hemagglutination activity of AIV incubated alone or with separate different substance in embryonated chicken eggs (ECE_S_)

Substance	Virus titer	Virus titer with compound
3	10^9^	10^1^
4		10^1^
5		10^1^
6		10^1^
8		10^5.6^
9		10^6.6^
10		10^6.6^
11		10^1^
13		10^4.8^
14		10^5.6^
15		10^5^
16		10^1^

Inhibition of hemagglutinating caused by virus is the detection of compound’s activity inhibition. Compounds **3, 5, 11, 6, 4,** and **16**, respectively, showed remarked antiviral activity, while compound **13** afforded less antiviral activity against avian influenza disease virus, followed by compounds **8, 10, 15, 14,** and **9** which showed little antiviral activity (Table 3). These results reflected that some products showing promising effect as anti-AI disease virus replication and offer great promise as potentially effective novel antiviral drugs.

HA titers of AIV-infected chicken embryos following treatment with different compounds are shown in Table 3. The antiviral assay revealed decreasing HA viral titer from the virus treated group to virus with the compound-treated group. Hemagglutination viruses are viruses capable of agglutinating the red blood cells of a variety of animals. They directly agglutinate erythrocytes by binding to specific receptor sites on the surface of the erythrocytes, and this characteristic can be used in detection of the virus. Inhibition of hemagglutinating caused by virus is the detection of compound’s activity inhibition. Compounds **3, 5, 11, 6, 4,** and **16,** respectively, showed remarked antiviral activity, while compound **13** afforded less antiviral activity against avian influenza disease virus, followed by compounds **8, 10, 15, 14,** and **9** which showed little antiviral activity (Table 3).

These results reflected that some products show promising effect as anti-AI disease virus replication and offer great promise as potentially effective novel antiviral drugs. Our obtained results come in accordance with the previous study [47] which investigated the antiviral effect of Curcuma zedoaria volatile oil and Hypericin perforatum liquid extract on H5N1 avian influenza virus (AIV) in MDCK cell line and non-AIV-immunized chickens, and attributed the virucidal activity to the presence of curcumenol and hypericin.

### Antimicrobial activity

Antimicrobial susceptibility of testing the 12 substances against two standard strains. The method includes the disk diffusion method as shown in Table 4. The examination of the antimicrobial activity data (Table 4) reveals that most of the compounds showed effective antibacterial and antifungal activity against the employed strains. Against the Gram-positive and the Gram-positive bacterium, compound has shown excellent activity.

**Table 4:** Antibacterial and antifungal activities of different compounds (diameter of inhibition zone in mm)

Compd	Inhibition diameter (mm)		MIC (µg/ml)		*Candida albicans*
	*Haemophilus* *(Gram –ve)*	*Staph* *(Gram* + *ve)*	*Haemophilus* *(Gram –ve)*	*Staph* *(Gram* + *ve)*	
**3**	22	23	1	2	24
**4**	22	25	6	5	25
**5**	23	24	3	3	22
**6**	25	22	5	4	20
**8**	4	8	12	13	8
**9**	1	2	20	19	2
**10**	6	7	17	13	7
**11**	22	25	2	5	24
**13**	12	14	10	9	14
**14**	8	7	16	16	7
**15**	7	6	16	18	6
**16**	23	22	6	5	23

The different compounds showed antimicrobial properties (Table 4) regarding their inhibition diameter ranging from 1 to 25 mm. The studied compounds played a positive role in boosting of immune system of the vaccinated chicks, reduction in clinical signs, and improvement in health status, and they reported as immune boosters. This was shown as comparing the humeral response of the vaccinated group (the first group vaccinated only) and other vaccinated groups which received the tested substance separately as immunostimulant reveals that compounds **3, 5, 11, 6, 4,** and **16** have special immune-boosting properties as they elevate the antibody titer in the serum of the vaccinated chicks. Followed by compounds **13, 8, 10, 15,** and **14**, on the other hand, compound **9** gave less immune-boosting properties (Table 5).

**Table 5:** Evaluation of immune-boosting properties of different substances in SPF chicks

Substance	Humeral response of vaccinated group (take vaccine only)	Humeral response of other vaccinated groups which received each tested compound separately
3	7 log2	7.3 log2
4		7.5 log2
5		7.4 log2
6		7.4 log2
8		6.6 log2
9		6log2
10		6.9 log2
11		7.1 log2
13		6.9 log2
14		7.2 log2
15		6.6 log2
16		7.2 log2

## Conclusion

The key material, 5-chloropyrazolyl-2(3*H*)-furanone derivative, has been obtained in a good yield via Perkin condensation of 5-chloro-4-formyl-3-methyl-1-phenylpyrazole with 3-(4-methylbenzoyl)propionic acid in the presence of cyclo-dehydrating agent. The titled furanone has been successfully converted into a new series of some pyrazole-based heterocycles such as pyrrolone, pyridazinone, and imidazole derivatives. Furthermore, some pyrrolones were achieved upon treating the acid hydrazide with various carbonyl reagents. The antioxidant activity assessments of some synthesized compounds showed that pyridazinone and *N*-phenylhydrazide derivatives were the most effective. The antimicrobial and antiviral activity screening shows the potential of using these compounds as antiviral agents and can be considered as a viable means to control the economically important avian influenza of poultry. These compounds can thus be recommended for their antiviral, antibacterial, and antifungal property and can very well be used as immunostimulants.

## Experimental

### General

All solvents and reagents were purified and dried by standard techniques. The reactions were monitored by thin-layer chromatography (TLC) using TLC sheets coated with UV fluorescent silica gel Merck 60 F254 plates and were visualized using UV lamp and different solvents as mobile phases. All melting points were measured on GALLENKAMP melting point apparatus and were uncorrected. The IR spectra were recorded on a Pye-Unicam SP-3-300 infrared spectrophotometer (KBr disks) and expressed in wave number (ν, cm^−1^). The ^1^H NMR and ^13^C NMR spectra were run at 400 and 100 MHz, on a Bruker Advance III NMR spectrometer, respectively (BRUKER, Manufacturing & Engineering Inc., Anaheim, CA, USA), at Faculty of Pharmacy, Cairo University. TMS was used as an internal standard using deuterated dimethyl sulfoxide (DMSO-*d*_*6*_) as a solvent. Chemical shifts (*δ*) are quoted in ppm. The mass spectra were recorded on a Shimadzu GCMS-QP-1000EX mass spectrometer at 70 eV. Elemental analyses were performed on a CHN analyzer, and all compounds were within ± 0.4 of the theoretical values. The starting pyrazole aldehyde **1** was previously reported [41].

#### 3-((5-Chloro-3-methyl-1-phenyl-1H-pyrazol-4-yl)methylene)-5-(p-tolyl)furan-2(3H)-one (3)

In a one-pot reaction, a mixture of 5-chloro-3-methyl-1-phenylpyrazole-4-carbaldehyde **(1)** (2.2 g, 0.01 mol), 4-oxo-4-(*p*-tolyl)butanoic acid (1.92 g, 0.01 mol) and freshly fused sodium acetate (0.82 g, 0.01 mol) in acetic anhydride (10 mL) was refluxed for 2 h. The solid obtained was collected and crystallized from ethanol to give orange crystals. Yield 65%, mp. 144–146 °C [48]. IR (KBr, cm^−1^): 1761 (C=O furanone). ^1^H NMR (DMSO-*d*_6_, *δ*, ppm): 7.73–7.09 (*m*, 11H, Ar–H + CH=), 2.41 (*s*, 3H, CH_3_ pyrazole), 2.36 (*s*, 3H, CH_3_ tolyl). ^13^C NMR (DMSO-*d*_6_, *δ*, ppm): 168.62, 155.30, 149.93, 141.6, 137.76, 130.09 (2), 129.78 (3), 129.33, 127.63 (3), 125.68, 125.61, 125.46, 123.73, 115.09, 101.71, 21.55, 14.04. MS (*m/z*, %): 376 (M˙^+^, 6), 342 (10), 275 (39), 212 (42), 185 (62), 171 (100), 160 (51), 115 (55), 97 (74), 58 (37). Anal. Calcd. for C_22_H_17_ClN_2_O_2_ (376.84): C, 70.12; H, 4.55; N, 7.43. Found: C, 70.01; H, 4.42; N, 7.46%.

#### 3-((5-Chloro-3-methyl-1-phenyl-1H-pyrazol-4-yl)methylene)-5-(p-tolyl)-1,3-dihydro-2H-pyrrol-2-one (4)

A mixture of **3** (3.77 g, 0.01 mol) and ammonium acetate (0.77 g, 0.01 mol) was heated in a sand bath at 130–140 °C for 5 h. The precipitate solid was filtered off and crystallized from dioxane to afford orange crystals. Yield 75%, mp. 216–218 °C [48]. IR (KBr, cm^−1^): 3179 (NH), 1689 (C=O). ^1^H NMR (DMSO-*d*_6_, *δ*, ppm): 10.50 (br.s, 1H, NH, exchangeable), 7.72–7.25 (*m*, 9H, Ar–H), 6.95 (*s*, 1H, CH=), 6.42 (*s*, 1H, C4-H pyrrolone), 2.41 (*s*, 3H, CH_3_ pyrazole), 2.34 (*s*, 3H, CH_3_ tolyl). ^13^C NMR (DMSO-*d*_6_, *δ*, ppm): 170.60, 149.52, 144.94, 139.3, 137.95, 132.51, 129.87, 129.78 (3), 129.11, 127.28, 126.77, 125.81 (3), 125.44, 118.36, 115.56, 97.98, 21.43, 14.10. MS (*m/z*, %): 375 (M˙^+^, 55), 348 (80), 319 (45), 279 (34), 259 (68), 168 (58), 148 (100), 117 (48), 58 (27), 41 (35). Anal. Calcd. for C_22_H_18_ClN_3_O (375.86): C, 70.30; H, 4.83; N, 11.18. Found: C, 70.17; H, 4.70; N, 11.15%.

#### N-Benzyl-2-((5-chloro-3-methyl-1-phenyl-1H-pyrazol-4-yl)methylene)-4-oxo-4-(p-tolyl)butanamide (5)

A solution of **3** (3.77 g, 0.01 mol) and benzylamine (1.7 mL, 0.01 mol) in benzene (20 mL) was refluxed for 3 h. The reaction mixture was filtered on hot and then recrystallized from benzene to afford **5** as white crystals. Yield 75%, mp. 118–120 °C [48]. IR (KBr, cm^−1^): 3287 (NH), 1668, 1641 (C=O). ^1^H NMR (DMSO-*d*_6_, *δ*, ppm): 7.57–7.10 (*m*, 15H, Ar–H + CH=), 6.77 (br.s, 1H, NH, exchangeable), 4.36 (*d*, 1H, Ha of NHCH_2_Ph), 4.08 (*d*, 1H, Hb of NHCH_2_Ph), 3.00 (*d*, 1H, Ha of CH_2_CO), 3.14 (*d*, 1H, Hb of CH_2_CO), 2.27 (*s*, 3H, CH_3_ pyrazole), 2.26 (*s*, 3H, CH_3_ tolyl). MS (*m/z*, %): 483 (M˙^+^, 15), 459 (12), 404 (13), 312 (31), 269 (34), 170 (27), 115 (68), 56 (37), 41 (100). Anal. Calcd. for C_29_H_26_ClN_3_O_2_ (484.00): C, 71.97; H, 5.41; N: 8.68. Found: C, 71.81; H, 5.28; N, 8.71%.

#### 2-((5-Chloro-3-methyl-1-phenyl-1H-pyrazol-4-yl)methylene)-N-isobutyl-4-oxo-4-(p-tolyl)butanamide (6)

A solution of **3** (3.77 g, 0.01 mol) and isobutylamine (0.73 mL, 0.01 mol) in benzene (20 mL) was heated under reflux for 3 h. The reaction mixture was cooled, and the formed solid was filtered off and recrystallized from benzene to furnish **6** as white crystals. Yield 83%, mp. 140–142 °C [48]. IR (KBr, cm^−1^): 3249 (NH), 1680, 1647 (C=O). ^1^H NMR (DMSO-*d*_6_, *δ*, ppm): 7.57–7.08 (*m*, 10H, Ar–H + CH=), 6.62 (br.s, 1H, NH, exchangeable), 3.22 (*s*, 2H, CH_2_CO), 3.12 (*d*, 2H, NHCH_2_,* J* = 7.5 Hz), 2.28 (*s*, 3H, CH_3_ pyrazole), 2.26 (*s*, 3H, CH_3_ tolyl), 1.78–1.72 (*m*, 1H, CH_2_CH), 0.76 (*d*, 6H, CH(CH_3_)_2_). MS (*m/z*, %): 449 (M˙^+^, 13), 426 (36), 373 (59), 324 (36), 267 (15), 240 (100), 203 (25), 134 (45), 122 (28), 90 (80), 43 (63). Anal. Calcd. for C_26_H_28_ClN_3_O_2_ (449.98): C, 69.40; H, 6.27; N, 9.34. Found: C, 69.24; H, 6.11; N, 9.30%.

#### 1-Benzyl-3-((5-chloro-3-methyl-1-phenyl-1H-pyrazol-4-yl)methylene)-5-(p-tolyl)-1,3-dihydro-2H-pyrrol-2-one (7)

A solution of pyrazole derivative **5** (4.83 g, 0.01 mol) in a mixture of hydrochloric/acetic acids (1:1) was refluxed for 2 h. The solid that separated on hot was washed several times with petroleum ether (60–80) to give **7** as yellow crystals. Yield 50%, mp. 152–154 °C [48]. IR (KBr, cm^−1^): 1698 (C=O). ^1^H NMR (DMSO-*d*_6_, *δ*, ppm): 7.64–7.03 (*m*, 15H, Ar–H + CH =), 6.16 (*s*, 1H, C4-H pyrrolone), 4.85 (*s*, 2H, CH_2_), 2.40 (*s*, 3H, CH_3_ pyrazole), 2.32 (*s*, 3H, CH_3_ tolyl). ^13^C NMR (DMSO-*d*_6_, *δ*, ppm): 169.81, 149.79, 148.29, 139.75, 138.30, 137.87, 130.18, 129.77 (2), 129.74, 129.20 (2), 128.98, 128.28 (2), 127.99, 127.48 (2), 127.12, 126.86 (2), 125.49 (2), 120.61, 115.27, 101.72, 44.33, 21.34, 14.11. Anal. Calcd. for C_29_H_24_ClN_3_O (465.98): C, 74.75; H, 5.19; N, 9.02. Found: C, 74.59; H, 5.02; N, 9.05%.

#### 2-((5-Chloro-3-methyl-1-phenyl-1H-pyrazol-4-yl)methylene)-4-oxo-4-(p-tolyl)butanehydrazide (8)

A solution of compound **3** (3.77 g, 0.01 mol) and hydrazine hydrate (1 mL, 0.02 mol, 80%) in ethanol (30 mL) was stirred for 30 min. The separated product was filtered off and crystallized from ethanol to produce **8** as white crystals. Yield 50%, mp. 142–144 °C [48]. IR (KBr, cm^−1^): 3309, 3263 (NH_2_, NH), 1704, 1677 (C = O). ^1^H NMR (DMSO-*d*_6_, *δ*, ppm): 7.57–7.10 (*m*, 10H, Ar–H + CH =), 6.60 (*br*.s, 1H, NH, exchangeable), 4.41 (br.s, 2H, NH_2_, exchangeable), 3.06 (*d*, 1H, Ha of CH_2_CO), 2.91 (*d*, 1H, Hb of CH_2_CO), 2.29 (*s*, 3H, CH_3_ pyrazole), 2.25 (*s*, 3H, CH_3_ tolyl). ^13^C NMR (DMSO-*d*_6_, *δ*, ppm): 166.23, 148.91, 140.87, 137.98, 137.13, 131.09, 129.67, (3) 129.01 (3), 125.94, 125.74 (2), 125.50 (2), 118.13, 114.47, 43.70, 21.08, 13.85. MS (*m/z*, %): 408 (M˙^+^, 13), 384 (5), 322 (11), 274 (6), 223 (84), 148 (16), 124 (33), 73 (41), 55 (48), 43 (100). Anal. Calcd. for C_22_H_21_ClN_4_O_2_ (408.89): C, 64.62; H, 5.18; N, 13.70. Found: C, 64.50; H, 5.04; N, 13.74%.

#### 4-((5-chloro-3-methyl-1-phenyl-1H-pyrazol-4-yl)methyl)-6-(p-tolyl)pyridazin-3(2H)-one (9)

A solution of furanone **3** (3.77 g, 0.01 mol) in ethanol (30 mL) was refluxed with hydrazine hydrate (1 mL, 0.02 mol, 80%) for 1 h. The product separated was filtered off and crystallized from dioxane to give **9** as off-white crystals, mp. 226–228 °C [48]. Yield 79%, IR (KBr, cm^−1^): 3203 (NH), 1655 (C=O). ^1^H NMR (DMSO-*d*_6_, *δ*, ppm): 13.17 (*br*.s, 1H, NH, exchangeable), 7.69–7.20 (*m*, 10H, Ar–H + CH pyridazine), 3.71 (*s*, 2H, CH_2_), 2.33 (*s*, 3H, CH_3_ pyrazole), 2.25 (*s*, 3H, CH_3_ tolyl). ^13^C NMR (DMSO-*d*_6_, *δ*, ppm): 168.93, 160.96, 149.31, 144.36, 140.80, 139.36, 139.17, 138.45, 132.63, 129.96, 129.58, 129.53, 129.45, 127.72, 126.07, 125.93, 125.79, 124.92, 113.31, 23.96, 21.25, 12.93. Anal. Calcd. for C_22_H_19_ClN_4_O (390.87): C, 67.60; H, 4.90; N, 14.33. Found: C, 67.42; H, 4.75; N, 14.35%.

#### 2-((5-Chloro-3-methyl-1-phenyl-1H-pyrazol-4-yl)methylene)-4-oxo-N'-phenyl-4-(p-tolyl)butanehydrazide (10)

A mixture of compound **3** (3.77 g, 0.01 mol) and phenylhydrazine (1.08 mL, 0.01 mol) in benzene (20 mL) was refluxed for 4 h (TLC). The solid product was filtered off, dried, and recrystallized from ethanol to give yellow crystals, mp. 174–176 °C [48]. Yield 72%, IR (KBr, cm^−1^): 3227, 3110 (NH), 1697, 1657 (C=O). ^1^H NMR (DMSO-*d*_6_, *δ*, ppm): 7.92 (*br*.s, 1H, NHCO, exchangeable), 7.59–6.70 (*m*, 15H, Ar–H + CH =), 6.68 (*br*.s, 1H, NHPh, exchangeable), 3.32 (*d*, 1H, Ha of CH_2_, *J* = 16 Hz), 3.14 (*d*, 1H, Hb of CH_2_, *J* = 16 Hz), 2.28 (*s*, 3H, CH_3_ pyrazole), 2.27 (*s*, 3H, CH_3_ tolyl). ^13^C NMR (DMSO-*d*_6_, *δ*, ppm): 166.84, 149.02, 140.78, 137.95, 137.26, 130.40, 129.69 (2), 129.08 (2), 128.78 (4), 126.10, 126.06 (2), 125.50 (3), 119.43, 119.09, 114.38, 113.48, 90.18, 43.69, 21.05, 13.94. MS (*m/z*, %): 484 (M˙^+^, 23), 472 (84), 429 (34), 395 (95), 374 (88), 348 (86), 311 (73), 199 (100), 175 (87), 137 (48), 84 (45), 43 (82). Anal. Calcd. for C_28_H_25_ClN_4_O_2_ (484.98): C, 69.34; H, 5.20; N, 11.55. Found: C, 69.19; H, 5.01; N, 11.50%.

#### 4-(5-Chloro-3-methyl-1-phenyl-1H-pyrazol-4-yl)-3-(4,5-dihydro-1H-imidazol-2-yl)-1-(p-tolyl)but-3-en-1-one (11)

A mixture of **3** (3.77 g, 0.01 mol) and ethylenediamine (0.6 mL, 0.01 mol) in ethanol and acetic acid glacial (0.1 mL) was refluxed for 4 h. The precipitate that separated was filtered off, dried, and washed with petroleum ether (80–100) to afford **11** as yellow crystals, mp. 202–204 °C [48]. Yield (74%), IR (KBr, cm^−1^): 3250 (NH), 1696 (C=O), 1652 (C=N). ^1^H NMR (DMSO-*d*_6_, *δ*, ppm): 7.55–7.06 (m, 10H, Ar–H + CH=), 6.60 (*br*.s, 1H, NH, exchangeable), 3.47 (*t*, 2H, = NCH_2_, *J* = 7.5 Hz), 3.09 (*s*, 2H, CH_2_CO), 2.83 (*t*, 2H, NHCH_2_, *J* = 7.4 Hz), 2.29 (*s*, 3H, CH_3_ pyrazole), 2.25 (*s*, 3H, CH_3_ tolyl). ^13^C NMR (DMSO-*d*_6_, *δ*, ppm): 167.61, 167.42, 148.93, 140.69, 140.67, 137.95, 137.40, 137.27, 132.40, 132.27, 129.67, 129.37, 129.23, 129.00, 125.77, 125.62, 125.46, 118.23, 114.58, 114.54, 89.58, 89.31, 45.26, 45.12, 21.12, 13.91, 13.87. MS (*m/z*, %): 418 (M˙^+^, 18), 404 (18), 344 (43), 307 (38), 235 (38), 169 (34), 115 (100), 77 (75), 68 (45), 41 (34). Anal. Calcd. for C_24_H_23_ClN_4_O (418.93): C, 68.81; H, 5.53; N, 13.37. Found: C, 68.70; H, 5.41; N, 13.40%.

#### N-(3-((5-Chloro-3-methyl-1-phenyl-1H-pyrazol-4-yl)methylene)-2-oxo-5-(p-tolyl)-2,3-dihydro-1H-pyrrol-1-yl)acetamide (12)

A suspension of hydrazide **8** (4.08 g, 0.01 mol) in acetic anhydride (10 mL) was stirred at room temperature for 1 h. The formed solid was filtered off and recrystallized from benzene to obtain yellow crystals, mp. 114–116 °C [48]. Yield 65%. IR (KBr, cm^−1^): 3235 (NH), 1724, 1681 (C=O), 1627 (C=N). ^1^H NMR (DMSO-*d*_6_, *δ*, ppm): 10.57 (*br*.s, 1H, NH, exchangeable), 7.63–7.11 (m, 10H, Ar–H + CH=), 6.30 (*s*, 1H, CH pyrrolone), 2.39 (*s*, 3H, CH_3_ pyrazole), 2.35 (*s*, 3H, CH_3_ tolyl), 1.88 (*s*, 3H, CH_3_CO). MS (*m/z*, %): 432 (M˙^+^, 24), 383 (50), 359 (40), 240 (48), 179 (66), 107 (51), 65 (100), 49 (44). Anal. Calcd. for C_24_H_21_ClN_4_O_2_ (432.91): C, 66.59; H, 4.89; N, 12.94. Found: C, 66.43; H, 4.77; N: 12.90%.

#### N-Acetyl-N-(3-((5-chloro-3-methyl-1-phenyl-1H-pyrazol-4-yl)methylene)-2-oxo-5-(p-tolyl)-2,3-dihydro-1H-pyrrol-1-yl)acetamide (13)

A suspension of the hydrazide **8** (4.08 g, 0.01 mol) in acetic anhydride (10 mL) was heated on water bath at 80–90 °C for 4 h. The reaction mixture was allowed to cool. The crude solid was filtered off, dried, and recrystallized from petroleum ether (60–80) to give red crystals, mp. 52–54 °C. Yield 60% [48]. IR (KBr, cm^−1^): 1734, 1690 (C=O), 1627 (C=N). ^1^H NMR (DMSO-*d*_6_, *δ*, ppm): 7.67–7.23 (m, 10H, Ar–H + CH=), 6.57 (*s*, 1H, CH pyrrolone), 2.42 (*s*, 3H, CH_3_ pyrazole), 2.35 (*s*, 9H, 3CH_3_). MS (*m/z*, %): 474 (M˙^+^, 12), 433 (50), 353 (44), 309 (22), 243 (55), 170 (28), 84 (100), 52 (91). Anal. Calcd. for C_26_H_23_ClN_4_O_3_ (474.95): C, 65.75; H, 4.88; N, 11.80. Found: C, 65.64; H, 4.68; N, 11.83%.

#### N-(3-((5-Chloro-3-methyl-1-phenyl-1H-pyrazol-4-yl)methylene)-2-oxo-5-(p-tolyl)-2,3-dihydro-1H-pyrrol-1-yl)benzamide (14)

A solution of hydrazide **8** (4.08 g, 0.01 mol) and benzoyl chloride (1.4 mL, 0.01 mol) in dry benzene (20 mL) was refluxed for 3 h. The reaction mixture was cooled, and the solid formed was filtered off, washed with light petroleum ether, dried, and then recrystallized from benzene to produce **14** as green crystals, mp. 310–312 °C [48]. Yield 60%. IR (KBr, cm^−1^): 3206 (NH), 1703, 1679 (C=O). ^1^H NMR (DMSO-*d*_6_, *δ*, ppm): 11.20 (*br*.s, 1H, NH, exchangeable), 7.52–7.07 (m, 15H, Ar–H + CH=), 5.84 (*s*, 1H, CH pyrrolone), 2.29 (*s*, 3H, CH_3_ pyrazole), 2.18 (*s*, 3H, CH_3_ tolyl). Anal. Calcd. for C_29_H_23_ClN_4_O_2_ (494.98): C, 70.37; H, 4.68; N, 11.32. Found: C, 70.21; H, 4.54; N, 11.29%.

#### 3-((5-Chloro-3-methyl-1-phenyl-1H-pyrazol-4-yl)methylene)-1-((4-chlorobenzylidene)-amino)-5-(p-tolyl)-1,3-dihydro-2H-pyrrol-2-one (15)

To a solution of **8** (4.08 g, 0.01 mol) in absolute ethanol (15 mL), 4-chlorobenzaldehyde (1.40 g, 0.01 mol) was added and the reaction mixture was refluxed for 3 h. The formed precipitate was filtered off and recrystallized from the dioxane to give **15** as orange crystals, mp. 190–192 °C [48]. Yield (80%). IR (KBr, cm^−1^): 1707 (C=O), 1632 (C=N). ^1^H NMR (DMSO-*d*_6_, *δ*, ppm): 9.49 (*s*, 1H, CH = N), 7.74–7.27 (*m*, 13H, Ar–H), 7.19 (*s*, 1H, CH =), 6.44 (*s*, 1H, CH pyrrolone), 2.41 (*s*, 3H, CH_3_ pyrazole), 2.36 (*s*, 3H, CH_3_ tolyl). ^13^C NMR (DMSO-*d*_6_, *δ*, ppm): 166.27, 153.07, 149.93, 146.75, 139.84, 137.82, 135.80, 133.71, 129.76 (3), 129.55 (3), 129.43, 129.37 (3), 129.29, 128.52 (2), 128.33, 127.62, 125.52, 121.70, 115.08, 101.02, 21.45, 14.11. MS (*m/z*, %): 512 (M˙^+^, 22), 493 (27), 457 (36), 411 (43), 375 (48), 258 (49), 242 (76), 186 (84), 165 (99), 118 (44), 91 (54), 65 (27), 44 (100). Anal. Calcd. for C_29_H_22_Cl_2_N_4_O (513.42): C, 67.84; H, 4.32; N, 10.91. Found: C, 67.73; H, 4.24; N, 10.95%.

#### 2-((5-Chloro-3-methyl-1-phenyl-1H-pyrazol-4-yl)methylene)-N'-((1,3-diphenyl-1H-pyrazol-4-yl)methylene)-4-oxo-4-(p-tolyl)butanehydrazide (16)

A mixture of **8** (4.08 g, 0.01 mol) and 1,3-diphenylpyrazole-4-carbaldehyde (2.48 g, 0.01 mol) in acetic acid was refluxed for 4 h. The formed precipitate was washed with petroleum ether (60–80), filtered off, and recrystallized from dioxane to give **16** as pale-buff crystals, mp. 182–184 °C [48]. Yield 65%, IR (KBr, cm^−1^): 3188 (NH), 1687 (C=O), 1645 (C=N). ^1^H NMR (DMSO-*d*_6_, *δ*, ppm): 8.87 (*s*, 1H, CH=N), 8.67 (*s*, 1H, C5-H pyrazole), 7.99 (br.s, 1H, NH, exchangeable), 7.56–7.27 (*m*, 20H, Ar–H + CH=), 3.29 (*d*, 1H, Ha of CH_2_, *J* = *Hz*), 3.06 (d, 1H, Hb of CH_2_, *J* = *Hz*), 2.32 (*s*, 3H, CH_3_ pyrazole), 2.28 (*s*, 3H, CH_3_ tolyl). ^13^C NMR (DMSO-*d*_6_, *δ*, ppm): 164.33, 152.20, 149.12, 145.01, 140.43, 139.43, 137.91, 137.62, 132.11, 130.01, 129.69 (3), 129.16 (4), 129.09 (4), 129.00 (4), 128.38 (4), 127.61, 127.55, 126.12, 125.53 (4), 125.33, 120.63, 119.41 (3), 117.46, 114.40, 90.51, 45.16, 21.11, 13.90. MS (*m/z*, %): 638 (M˙^+^, 12), 549 (18), 477 (18), 338 (25), 310 (100), 230 (63), 171 (92), 143 (48), 74 (65), 43 (94). Anal. Calcd. for C_38_H_31_ClN_6_O_2_ (639.16): C, 71.41; H, 4.89; N, 13.15. Found: C, 71.24; H, 4.81; N, 13.11%.

#### Determination of total antioxidant capacity (TAC)

The antioxidant activity of each compound was recorded according to phosphomolybdenum method using ascorbic acid as standard [43]. This assay was based on the reduction of Mo (VI) to Mo (V) by the sample analyte and subsequent formation of a green-colored [phosphate = Mo (V)] complex at acidic pH with a maximal absorption at 695 nm. In this method, 0.5 ml of each compound (500 µg/ml) in methanol was combined in dried vials with 5 ml of reagent solution (0.6 M sulfuric acid, 28 mM sodium phosphate, and 4 mM ammonium molybdate). The vials containing the reaction mixture were capped and incubated in a thermal block at 95 °C for 90 min. After cooling the samples to room temperature, the absorbance was measured at 695 nm against a blank. The blank consisted of all reagents and solvents without the sample, and it was incubated under the same conditions. All experiments were carried out in triplicate. The antioxidant activity of the sample was expressed as the number of ascorbic acid equivalent (AAE) [44].

### Statistical analysis

All data were presented as mean ± SD using SPSS 13.0 program (SPSS Inc. USA).

#### Antiviral activity

*Avian influenza virus* The HPAI-H5N1 isolate clade (OP491851.1) was used which has accession number (A/ibis/Egypt/RLQP-229S/2022 (H5N1) segment4 hemagglutination (HA) gene.

#### Embryonated specific pathogen-free (SPF) chicken eggs (ECES)

One-day-old specific pathogen-free (SPF) 780 eggs were obtained from the National Project for production of specific pathogen-free eggs, Kom Oshim, Fayoum, Egypt. They were kept in the egg incubator at 37 °C with humidity 60–80% till the age of 9–11 days.

#### Chicken erythrocytes

Freshly collected chicken erythrocytes (1% and 10%) prepared in saline solution after several washes were used in the hemagglutination assay (HA).

#### Bacterial materials

Standard strains of *Staphylococcus aureus* and *Haemophilus* were obtained from bacterial strain bank of the Central Laboratory for Evaluation of Veterinary Biologics (CLEVB).

#### In vitro study

Screening of 12 substances antiviral activity must be done using non-cytotoxic concentration of it. Therefore, evaluation of the substances as inhibitory agents against AIV replication in SPF chicken embryos and their cytotoxicity was carried out.

#### Cytotoxicity (CC_50_)

Nontoxic concentrations of the tested substance (lower than CC_50_) were checked for antiviral properties against AIV replication in SPF chicken embryos. Groups of 9–11 days of embryonated chicken eggs (SPF) were inoculated with different concentrations of each tested substance separately for calculation of cytotoxicity concentration fifty (CC50). Un-inoculated SPF eggs were always included as control of embryo. The eggs were inoculated via allantoic cavity and were incubated for 6 days PI at 37 °C with humidity 60–80%. Cytotoxicity concentration fifty (CC_50_) of each test compound was determined as the concentration of compound that induced any embryos mortalities or any deviation than normal control embryos in 50% of embryonated chicken eggs.

#### Inhibition concentration (IC_50_)

Other groups of (SPF) embryonated chicken eggs were inoculated with a mixture of minimal cytotoxic concentration of different tested substances separately with 10^9^EID50 /mL of AIV (0.2 mL per egg) for calculation of the antiviral inhibitory concentration fifty (IC_50_). Un-inoculated SPF eggs were always included as control of embryo. The eggs were inoculated via allantoic cavity and were incubated for 6 days PI at 37 °C with humidity 60–80%. The antiviral inhibitory concentration fifty (IC_50_) of tested compounds was assayed as the concentration of the compound that fully inhibited the virus effect in 50% of embryonated chicken eggs.

*The therapeutic index (TI)* of the tested substances was expressed as CC_50_/IC_50_ and calculated using the method of Reed and Muench 1938.

#### In vitro antiviral assay

- The study was carried out in ovo in 9-day-old embryonated chicken eggs (ECE) according to the procedure mentioned with slight modifications. Stock solutions were prepared from the working solution by dissolving 0.5 g of the compound in 5 mL of dimethyl sulfoxide (DMSO) to make 10% w/v working solutions. Then, procedures are done as follows:
- Twelve-fold serial dilutions of the AIV were performed.
- 0.2 mL of each substance was added separately and incubated for 1 h at room temperature.
- Groups (eggs) of 9–11-day-old specific pathogen-free (SPF) were inoculated by varying serial dilutions (from 1st dilution to 12th dilution each dilution in five eggs) of each mixture (0.2 mL per egg) which were inoculated via allantoic route.
- By daily examination of the inoculated eggs, deaths within 24 h post-inoculation (PI) were discarded, and mortality between days 2 and 6 post-inoculation considered being specific.
- The AIV infectivity in ECE was determined by the hemagglutinating activity of the allantoic fluid of the inoculating eggs as measured by the microtechnique of the hemagglutinating (HA) test [49].
- Virus titer was calculated using the method [50].
- The known AIV titer was compared with the antiviral activities.

#### Methodology for in vitro antimicrobial screening or MIC measurement

The in vitro antimicrobial activities of all the compounds were assessed against Gram-positive bacteria, Gram-negative bacteria, and Candida albicans fungus by using agar diffusion method [51]. The treated and the controls were kept in an incubator at 37 °C for bacterial strains and at 28 °C for 24–72 h. The zones of inhibition for each well were measured. Nystatin, erythromycin, oxacillin, methicillin, and bacitracin were included in the test as references. At the end of incubation period, the diameter (mm) of the inhibition zones was measured.

The stock solutions of the different compounds were diluted and transferred into the first tube, and serial dilutions were performed so that concentrations in the range of 0.001–0.02 µL/mL were obtained. A 10-µL spore suspension of each test strain was inoculated in the test tubes in nutrient medium and incubated at 37 °C for 24–72 h. The control tubes containing the same medium were inoculated only with bacterial strains suspension. The minimal concentrations at which no visible growth was observed were defined as the MICs which were expressed in (v/v %).

Bacterial strains were primarily inoculated into Mueller–Hinton agar, and after overnight growth, a number of colonies were directly suspended in saline solution until the turbidity matched the turbidity of the McFarland standard (approximately 10^8^ CFU/mL), i.e., the inoculum size for the test strain was adjusted to 10^8^ CFU/mL by comparing the turbidity (turbidimetric method). Similarly, fungi were inoculated in Sabouraud dextrose broth, and the procedures of inoculum standardization were also similar. DMSO was used as diluent/vehicle to get the desired concentration of synthesized compounds to test the standard microbial strains, i.e., the compounds were dissolved in DMSO, and the solutions were diluted with culture medium. The interpretation of the results was based on nystatin, erythromycin, oxacillin, methicillin, and bacitracin.

## Supplementary Information

Below is the link to the electronic supplementary material.

Supplementary file1 (DOCX 6012 kb)

## Data Availability

*Supporting Information* Full spectroscopic data can be found via “Supplementary Content” section of this article’s webpage.

## References

[1] El-Helw EAE, Gado MM, El-Ziaty AK (2020). Synthesis and anti-rotavirus activity of some nitrogen heterocycles integrated with pyrazole scaffold. J. Iran. Chem. Soc..

[2] Kaddah MM, Fahmi AA, Kamel MM, Ramadan SK, Rizk SA (2021). Synthesis, characterization, computational chemical studies and antiproliferative activity of some heterocyclic systems derived from 3-(3-(1,3-diphenyl-1H-pyrazol-4-yl)acryloyl)-2*H*-chromen-2-one. Synth. Commun..

[3] Ramadan SK, Abou-Elmagd WSI (2018). Synthesis and anti H_5_N_1_ activities of some novel fused heterocycles bearing pyrazolyl moiety. Synth. Commun..

[4] El-Badawy AA, Elgubbi AS, El-Helw EAE (2021). Acryloyl isothiocyanate skeleton as a precursor for synthesis of some novel pyrimidine, triazole, triazepine, thiadiazolopyrimidine and acylthiourea derivatives as antioxidant agents. J. Sulfur Chem..

[5] Kaddah MM, Morsy AR, Fahmi AA, Rizk SA, Ramadan SK (2021). Synthesis and biological activity on IBD virus of diverse heterocyclic systems derived from 2-cyano-*N*'-((2-oxo-1,2-dihydroquinolin-3-yl)methylene)acetohydrazide. Synth. Commun..

[6] Halim KN, Rizk SA, El-Hashash MA, Ramadan SK (2021). Straightforward synthesis, antiproliferative screening, and density functional theory study of some pyrazolylpyrimidine derivatives. J. Heterocycl. Chem..

[7] Ramadan SK, El-Ziaty AK, El-Helw EAE (2021). Synthesis and antioxidant evaluation of some heterocyclic candidates from 3-(1,3-diphenyl-1*H*-pyrazol-4-yl)-2-(4-oxo-4*H*-benzo[*d*][1,3]oxazin-2-yl)propenonitrile. Synth. Commun..

[8] Sallam HA, Elgubbi AS, El-Helw EAE (2020). Synthesis and antioxidant screening of new 2-cyano-3-(1,3-diphenyl-1H-pyrazol-4-yl)acryloyl amide derivatives and some pyrazole-based heterocycles. Synth. Commun..

[9] Ramadan SK, Sallam HA (1942). Synthesis, spectral characterization, cytotoxic, and antimicrobial activities of some novel heterocycles utilizing 1,3-diphenylpyrazole-4-carboxaldehyde thiosemicarbazone. J. Heterocycl. Chem..

[10] El-Helw EAE, Morsy AR, Hashem AI (2021). Evaluation of some new heterocycles bearing 2-oxoquinolyl moiety as immunomodulator against highly pathogenic avian influenza virus (H_5_N_8_). J. Heterocycl. Chem..

[11] El-Helw EAE, Hashem AI (2020). Synthesis and antitumor activity evaluation of some pyrrolone and pyridazinone heterocycles derived from 3-((2-oxo-5-(p-tolyl)furan-3(2H)-ylidene)methyl)quinolin-2(1H)-one. Synth. Commun..

[12] Ramadan SK, Haleem DR, Abd-Rabboh HS, Gad NM, Abou-Elmagd WSI, Haneen DS (2022). Synthesis, SAR studies, and insecticidal activities of certain N-heterocycles derived from 3-((2-chloroquinolin-3-yl)methylene)-5-phenylfuran-2(3H)-one against *Culex pipiens* L. larvae. RSC Adv..

[13] Ramadan SK, Abd-Rabboh HSM, Gad NM, Abou-Elmagd WSI, Haneen DSA (2022). Synthesis and characterization of some chitosan-quinoline nanocomposites as potential insecticidal agents. Polycycl. Arom. Compds..

[14] Morsy AR, Ramadan SK, Elsafty MM (2020). Synthesis and antiviral activity of some pyrrolonyl substituted heterocycles as additives to enhance inactivated Newcastle disease vaccine. Med. Chem. Res..

[15] Abbas SH, Abuo-Rahma GA, Abdel-Aziz M, Aly OM, Beshr EA, Gamal-Eldeen AM (2016). Synthesis, cytotoxic activity, and tubulin polymerization inhibitory activity of new pyrrol-2(3*H*)-ones and pyridazin-3(2*H*)-ones. Bioorg. Chem..

[16] Gilchrist, T.L. Pitman Publishing Ld, London WC2E9AN. **1985**, 195, 127.

[17] Ramadan SK, Abou-Elmagd WSI, Hashem AI (2019). Reactions of 2(3*H*)-furanones. Synth. Commun..

[18] Sawant P, Maier Martin E (2010). Synthesis of atorvastatin lactone linker constructs for target fishing. Tetrahedron.

[19] Gad NM, Abou-Elmagd WSI, Haneen DSA, Ramadan SK (2021). Reactivity of 5-phenyl-3-[(2-chloroquinolin-3-yl)methylene] furan-2(3H)-one towards hydrazine and benzylamine: a comparative study. Synth. Commun..

[20] Valko M, Izakovic M, Mazur M, Rhodes CJ, Telser J (2004). Role of oxygen radicals in DNA damage and cancer incidence. Mol. Cell. Biochem..

[21] Lobo V, Patil A, Phatak A, Chandra N (2010). Free radicals, antioxidants and functional foods: impact on human health. Pharmacogn. Rev..

[22] FAO. Faostat. Production. Live animals. Rome, Italy. Accessed Jan 2022 (2012). https://www.fao.org/faostat/en/#home

[23] A. Pemin, G. Pedersen, J.C. Riise. Poultry as a tool for poverty alleviation: opportunities and problems related to poultry production at village level. Pages 143–147 in ACIAR proceedings (2001).

[24] Khan HM, Raza SM, Anjum AA, Ali MA (2019). Antiviral, embryo toxic and cytotoxic activities of *Astragalus membranaceus* root extracts. Pak. J. Pharm. Sci..

[25] Tong S, Li Y, Rivailler P, Conrardy C, Castillo DA, Chen LM, Recuenco S, Ellison JA, Davis CT, York IA, Turmelle AS, Moran D, Rogers S, Shi M, Tao Y, Weil MR, Tang K, Rowe LA, Sammons S, Xu X, Frace M, Lindblade KA, Cox NJ, Anderson LJ, Rupprecht CE, Donis RO (2012). A distinct lineage of influenza A virus from bats. Proc. Natl. Acad. Sci. U.S.A..

[26] Pantin-Jackwood MJ, Swayne DE (2007). Pathobiology of Asian highly pathogenic avian influenza H5N1 virus infections in ducks. Avian Dis..

[27] Lee J-Y, Abundo MEC, Lee C-W (2018). Herbal medicines with antiviral activity against the influenza virus, a systematic review. Am. J. Chin. Med..

[28] Bai Y, Jones JC, Wong S-S, Zanin M (2021). Antivirals targeting the surface glycoproteins of influenza virus: mechanisms of action and resistance. Viruses.

[29] Masalha M, Borovok I, Schreiber R, Aharonowitz Y, Cohen G (2001). Analysis of transcription of the Staphylococcus aureus aerobic class Ib and anaerobic class III ribonucleotide reductase genes in response to oxygen. J. Bacteriol..

[30] El-Sayed AA, Ismail MF, Amr AE, Naglah AM (2019). Synthesis, antiproliferative, and antioxidant evaluation of 2-pentylquinazolin-4(3h)-one(thione) derivatives with DFT study. Molecules.

[31] Youssef YM, Azab ME, Elsayed GA, El-Sayed AA, El-Helw EAE (2022). Synthesis and antiproliferative screening of some heterocycles derived from 4-((5-chloro-3-methyl-1-phenyl-1h-pyrazol-4-yl)methylene)-2-phenyloxazol-5(4h)-one. Polycycl. Arom. Compd..

[32] Hemdan MM, Fahmy AFM, Aly NF, Hegazi IA, El-Sayed AA (2012). Utility of phthalimidoacyl isothiocyanate in synthesis of quinazolines, benzoxazoles, benzimidazoles, 1,2,4-triazoles, and oxatriazepines. Phosph. Sulfur Silicon Rel. Elem.

[33] Rizk SA, El-Sayed AA, Mounier MM (2017). Synthesis of novel pyrazole derivatives as antineoplastic agent. J. Heterocycl. Chem..

[34] Hemdan MM, El-Sayed AA (2016). Use of Phthalimidoacetyl isothiocyanate as a scaffold in the synthesis of target heterocyclic systems, and their antimicrobial assessment. Chem. Pharm. Bull..

[35] Salem MS, El-Helw EAE, Derbala HA (2020). Development of chromone-pyrazole-based anticancer agents. Russ. J. Bioorg. Chem..

[36] Ghareeb EA, Mahmoud NFH, El-Bordany EA, El-Helw EAE (2021). Synthesis, DFT, and eco-friendly insecticidal activity of some *N*-heterocycles derived from 4-((2-oxo-1,2-dihydroquinolin-3-yl)methylene)-2-phenyloxazol-5(4H)-one. Bioorg. Chem..

[37] El-Naggar AM, Ramadan SK (2020). Efficient synthesis of some pyrimidine and thiazolidine derivatives bearing quinoline scaffold under microwave irradiation. Synth. Commun..

[38] Ramadan SK, Ibrahim NA, El-Kaed SA, El-Helw EAE (2021). New potential fungicides pyrazole-based heterocycles derived from 2-cyano-3-(1,3-diphenyl-1*H*-pyrazol-4-yl) acryloyl isothiocyanate. J. Sulfur Chem..

[39] Abdelrahman AM, Fahmi AA, Rizk SA, El-Helw EAE (2021). Synthesis, DFT and antitumor activity screening of some new heterocycles derived from 2,2'-(2-(1,3-diphenyl-1*h*-pyrazol-4-yl)ethene-1,1-diyl)bis(4*h*-benzo[*d*][1,3]oxazin-4-one). Polycycl. Arom. Compd..

[40] Hassaballah AI, Ramadan SK, Rizk SA, El-Helw EAE, Abdelwahab SS (2022). Ultrasonic promoted regioselective reactions of the novel spiro 3,1-benzoxazon-isobenzofuranone dye toward some organic base reagents. Polycycl. Aromat. Compd..

[41] Xu C-J, Shi Y-Q (1816). Synthesis and crystal structure of 5-chloro-3-methyl-1-phenyl-1h-pyrazole-4-carbaldehyde. J. Chem. Crystallogr..

[42] P.M. Brown, J. Russell, R.H. Thomsoon, A.G. Wylile. J. Chem. Soc. (c), **1968**, 842.

[43] Prieto P, Pineda M, Aguilar M (1999). Spectrophotometric quantation of antioxidant capacity through the formation of a phosphomolybdenum complex: specific application to the determination of vitamin E. Anal. Biochem..

[44] Ostby OB, Gundersen LL, Rise F, Antonsen O, Fosnes K, Larsen V, Bast A, Custers I, Haenen GR (2001). Synthesis of 5-substituted pyrrolo[1,2-*b*]pyridazines with antioxidant properties. Arch. Pharm. (Weinheim).

[45] Rice-Evance CA, Miller NJ, Paganga G (1996). Structure-antioxidant activity relationships of flavonoids and phenolic acids. Free Radic. Biol. Med..

[46] Imada I, Sato EF, Miyamoto M, Ichimori Y, Minamiyama Y, Konaka R, Inoue M (1999). Analysis of reactive oxygen species generated by neutrophils using a chemiluminescence probe L-012. Anal. Biochem..

[47] YaDong H, YanMei L, Qi X, ZhiJian S, LinChuan W, XiaoKun L, ZhiFeng H (2009). Antiviral effect on H5N1 avian influenza virus of compound of *Curcuma zedoaria* volatile oil and *Hypericin perforatum* extract liquid. J. China Pharma Univ..

[48] Youssef YM, Azab ME, Elsayed GA, El-Sayed AA, Hassaballah AI, El-Helw EAE (2023). Synthesis and antioxidant activity of some pyrazole-based heterocycles using a 2(3H)-furanone building block. Synth. Commun..

[49] Takasty GX (1996). The use of spiral loops in serological and virological method. Aceta Microbial. Hung..

[50] Reed LJ, Muench H (1938). A simple method of estimating fifty percent end point. Am. J. Hyg..

[51] Linday EM (1962). Practical Introduction to Microbiology.

