# Supplementary material for: Synthesis and antioxidant, antimicrobial, and antiviral activity of some pyrazole-based heterocycles using a 2(3H)-furanone derivative
Source: J IRAN CHEM SOC. 2023 Jun 3:1–14. Online ahead of print. doi: 10.1007/s13738-023-02814-w (PMC10239048; doi:10.1007/s13738-023-02814-w)
Supplement: Supplementary file 1 — Supplementary file1 (DOCX 6012 kb) [file 13738_2023_2814_MOESM1_ESM.docx]

**Synthesis, Antioxidant, Antimicrobial, and Antiviral Activity of Some Pyrazole-based Heterocycles Using A 2(3*H*)-Furanone Derivative**

**Youssef M. Youssef ^a^, Mohammad E. Azab ^a^, Galal A. Elsayed ^a^, Amira A. El-Sayed ^a^, Aya I. Hassaballah ^a^, Mounir M. El-Safty ^b^, Reem A. Soliman ^c^, Eman A. E. El-Helw ^a,^***

^a^ Chemistry Department, Faculty of Science, Ain Shams University, Cairo 11566, Egypt

^b^ Chief Researcher Department of Evaluation of Inactivated Viral Poultry Vaccines, Department of Quality Control of SPF Eggs, Central Laboratory for Evaluation of Veterinary Biologics, Agriculture Research Center (ARC), Egypt.

^c^ Researcher in Department of Evaluation of Inactivated Viral Poultry Vaccines, Department of Quality Control of SPF Eggs, Central Laboratory for Evaluation of Veterinary Biologics, Agriculture Research Center (ARC), Egypt.

**E*-mail: [eman.abdelrahman@sci.asu.edu.eg](mailto:eman.abdelrahman@sci.asu.edu.eg)

**Supporting information**


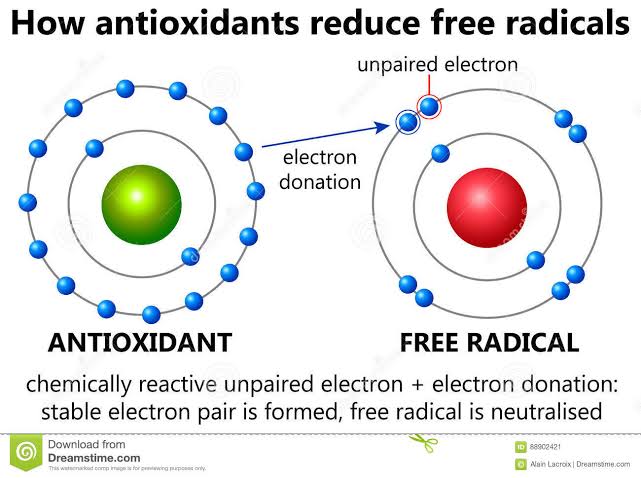


**Fig. 2**. Importance of antioxidants.

**Spectral Data**


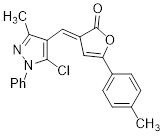

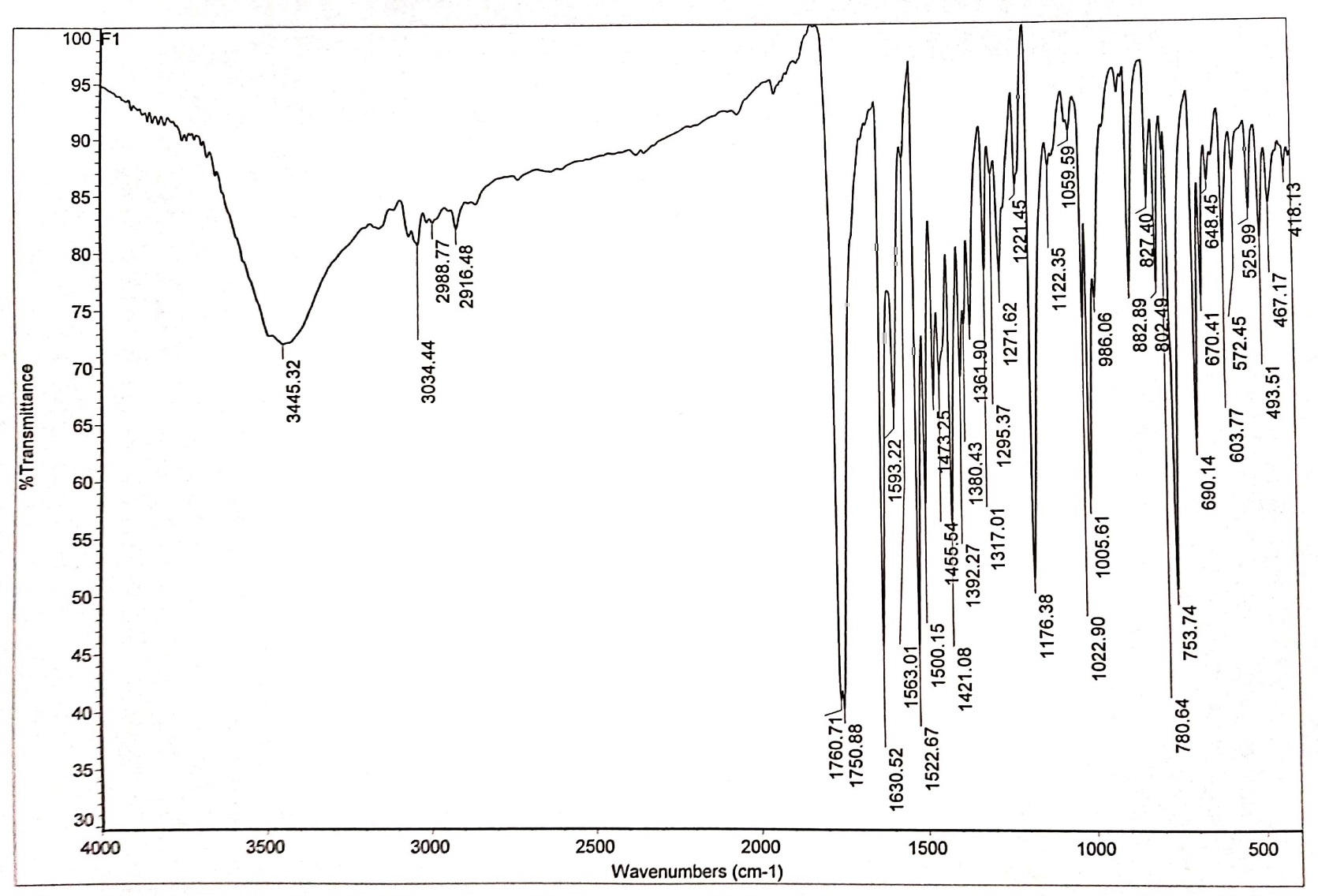


**Fig. 1: IR spectrum of compound (3)**


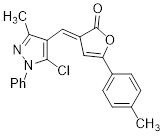

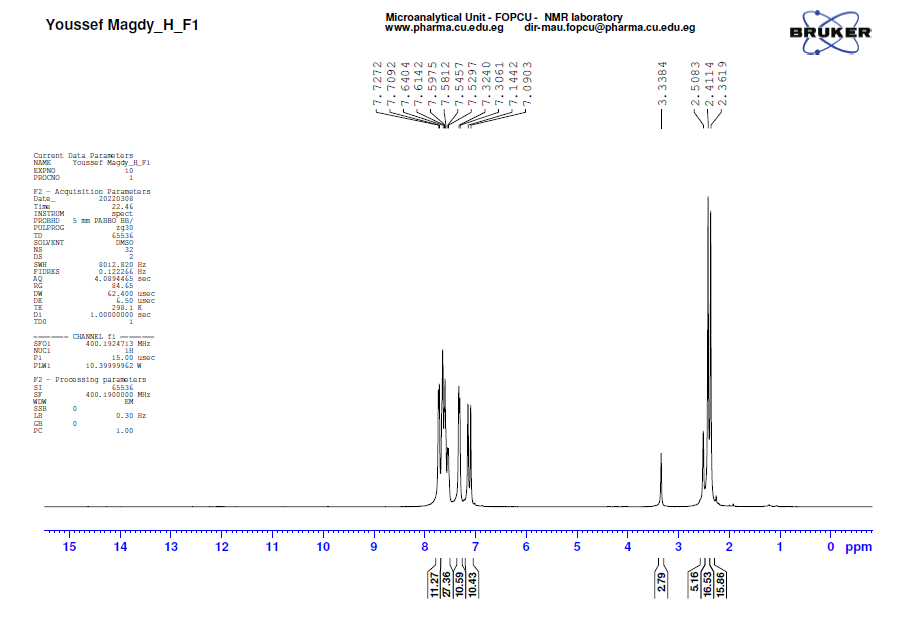

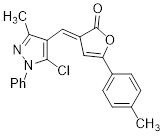

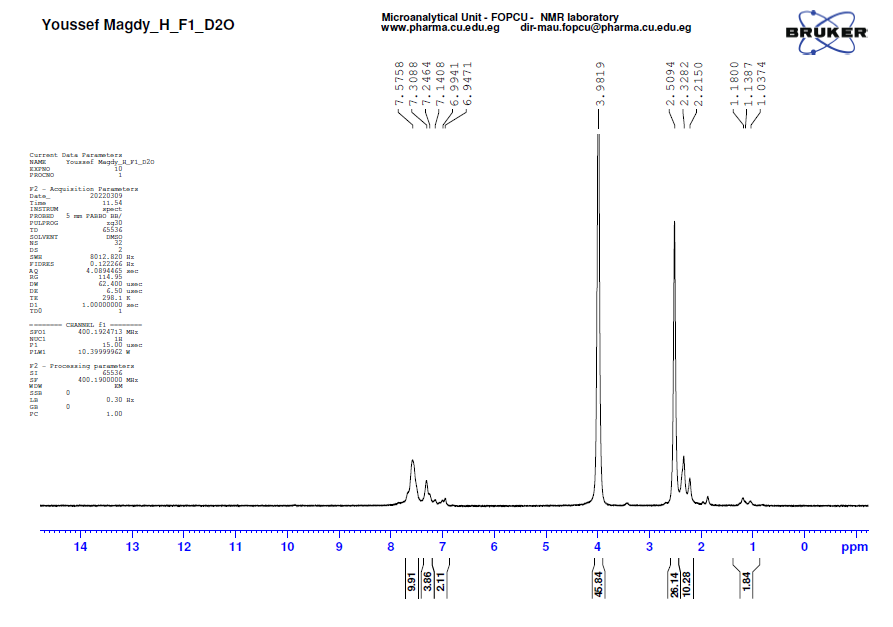


**Fig. 2: ^1^H NMR spectrum of compound (3)**

**Fig. 3: ^1^H NMR-D_2_O spectrum of compound (3)**


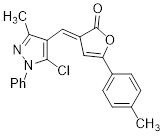

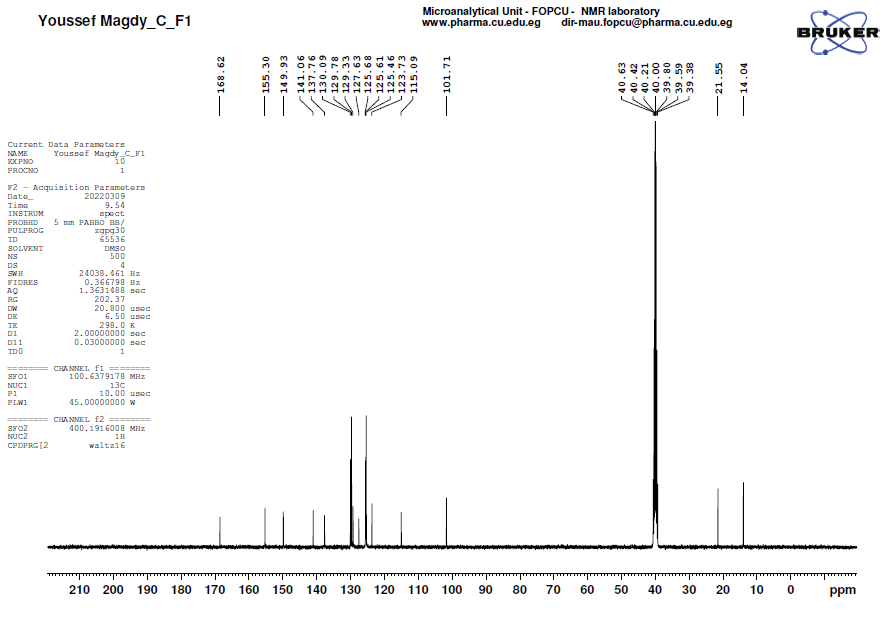


**Fig. 4: ^13^C NMR spectrum of compound (3)**


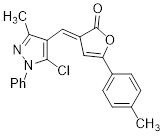


**Fig. 5: Mass spectrum of compound (3)**


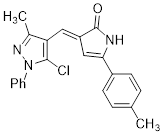

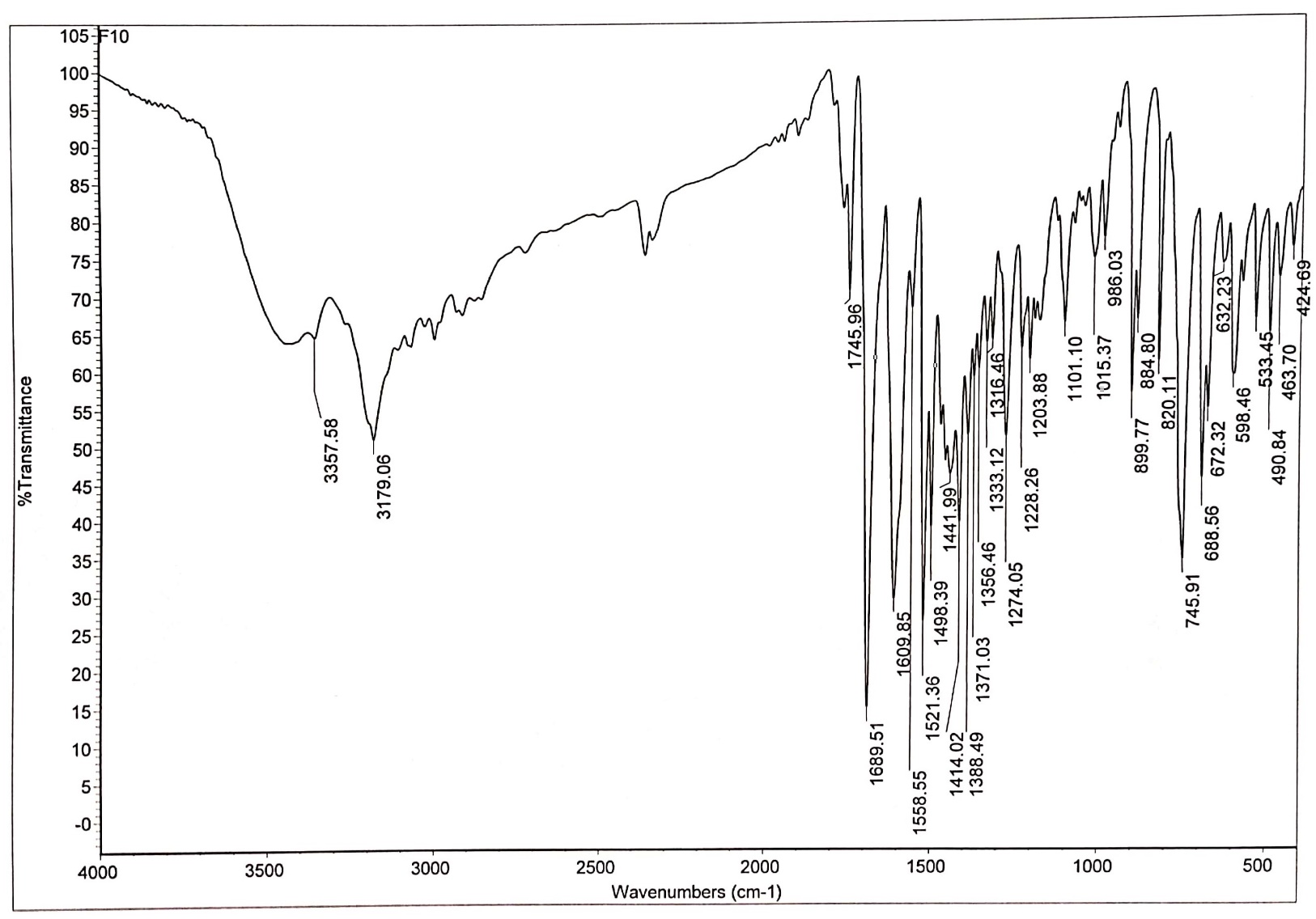


**Fig. 6: IR spectrum of compound (4)**


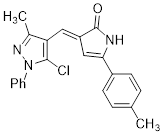

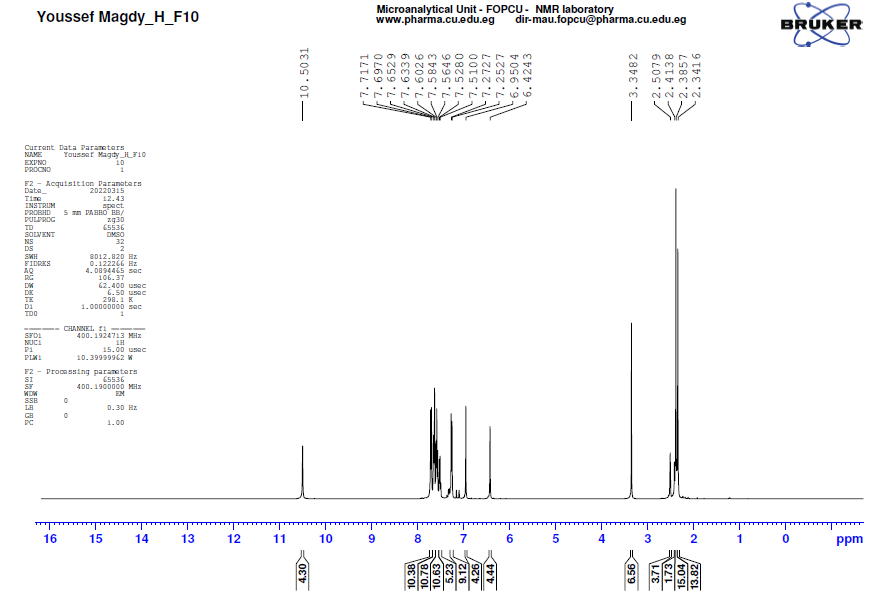

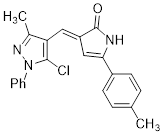

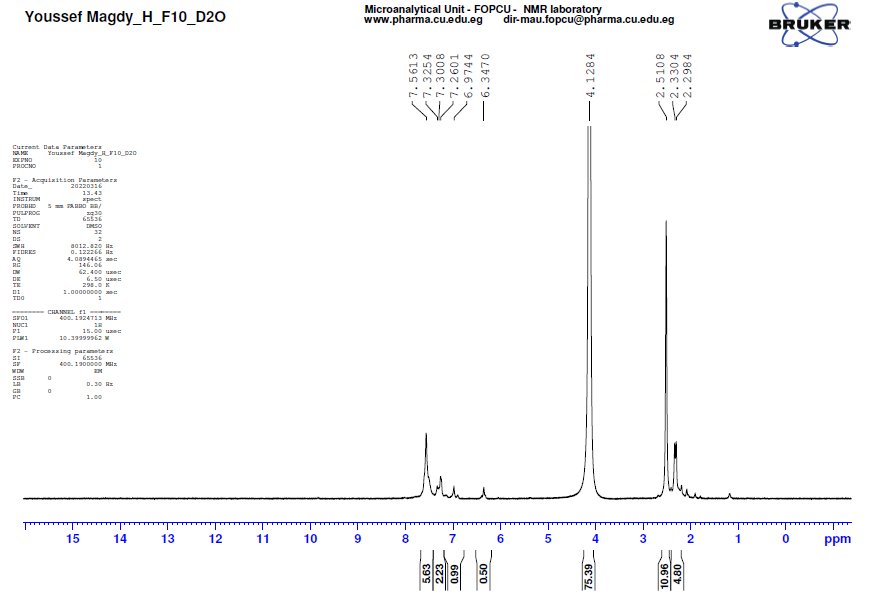

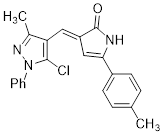

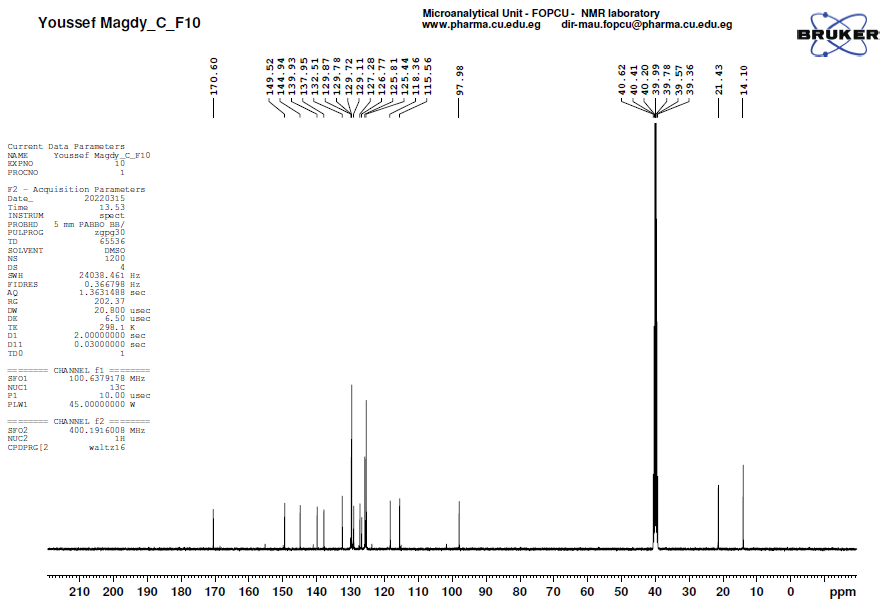

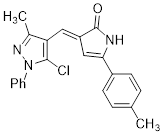

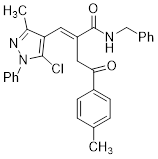

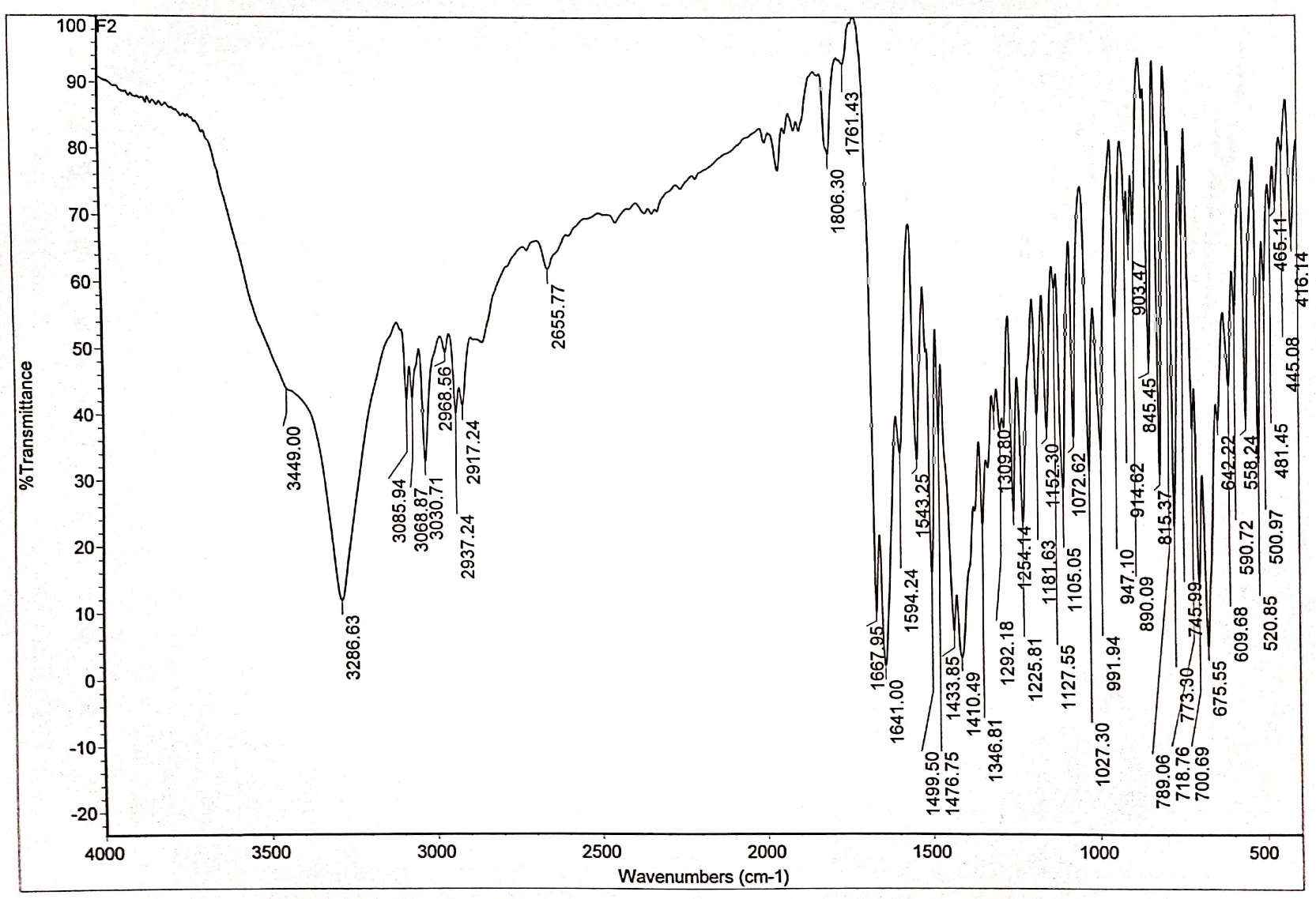

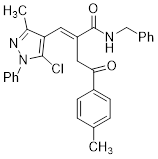

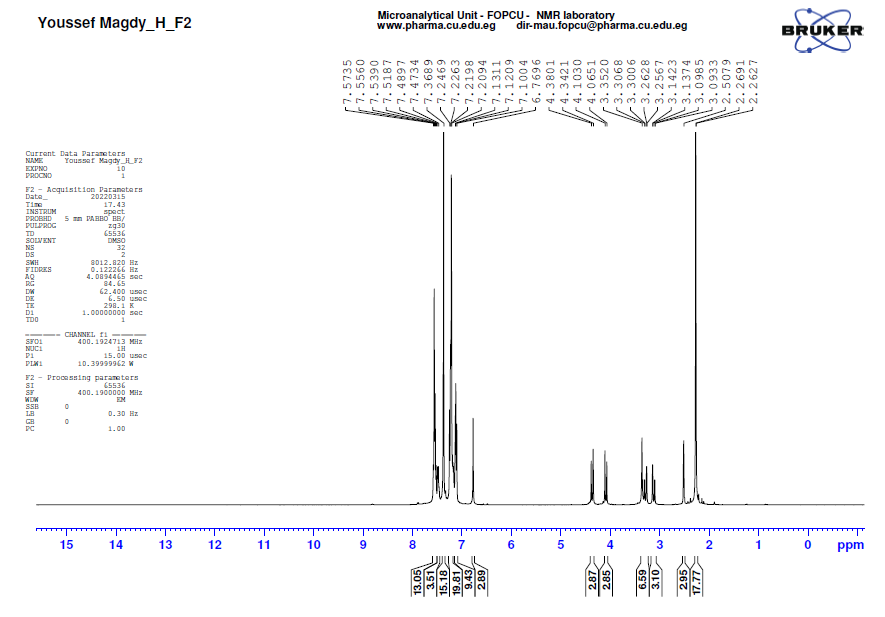

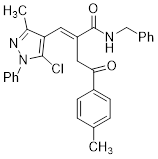

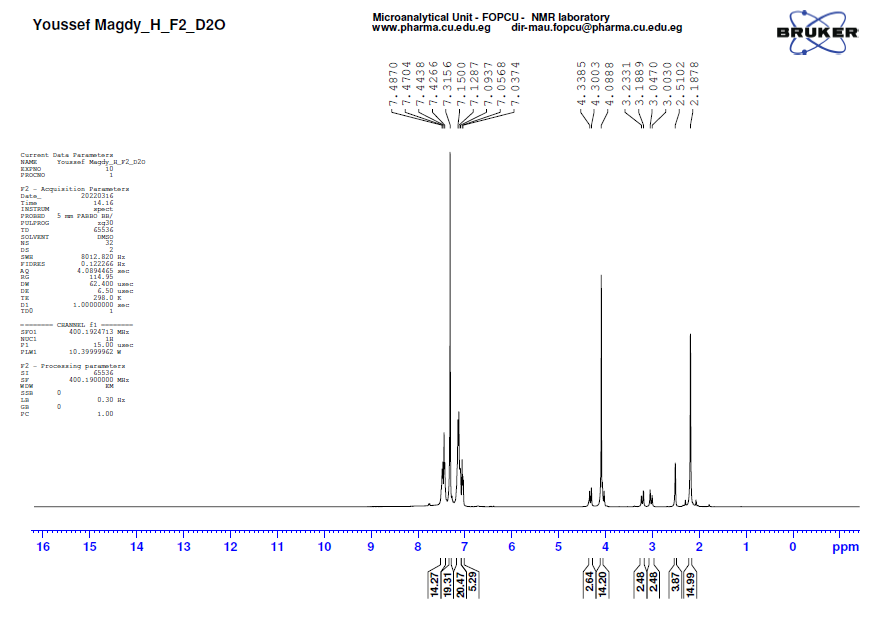

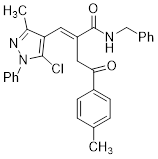

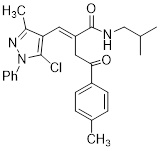

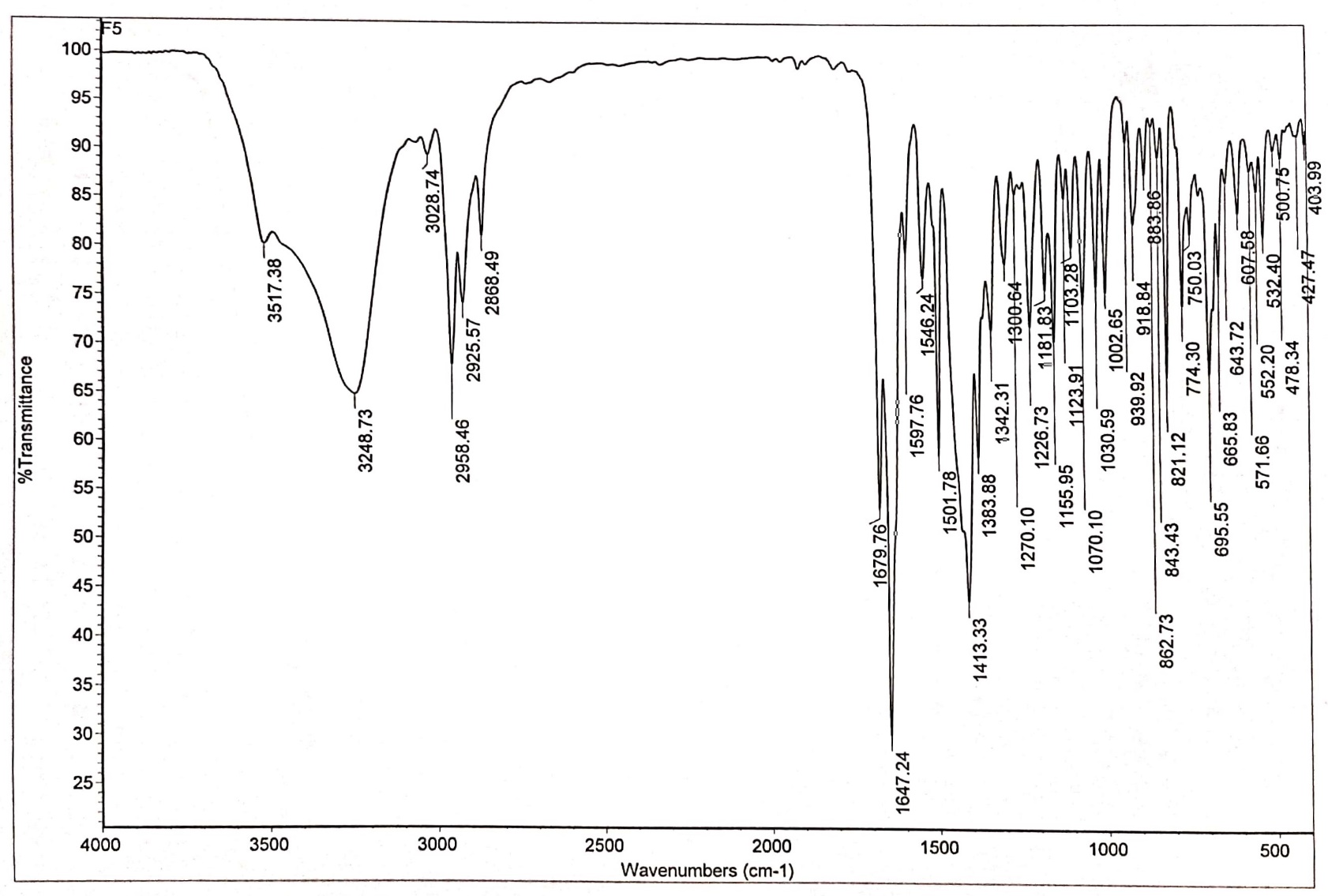

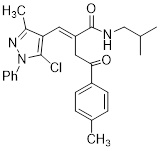

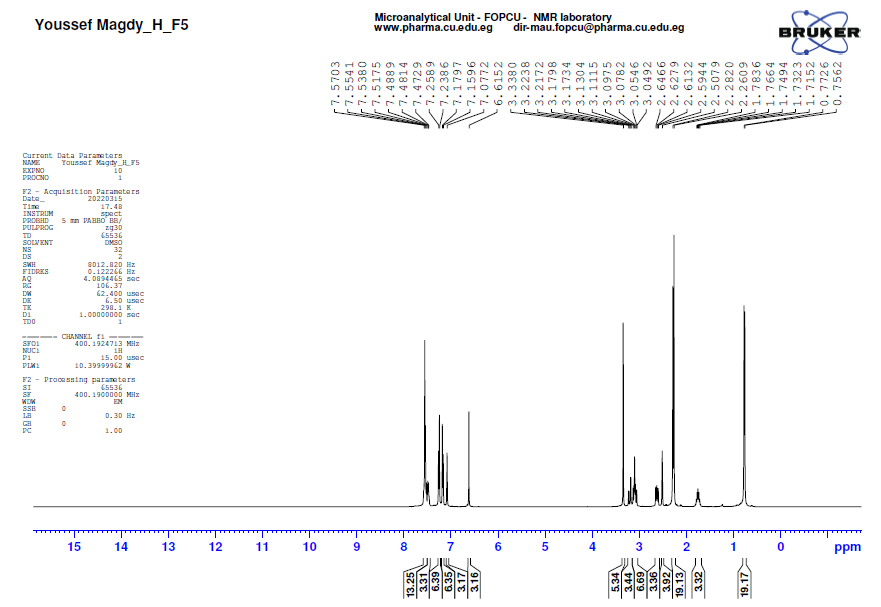

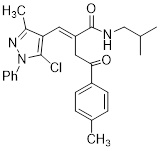

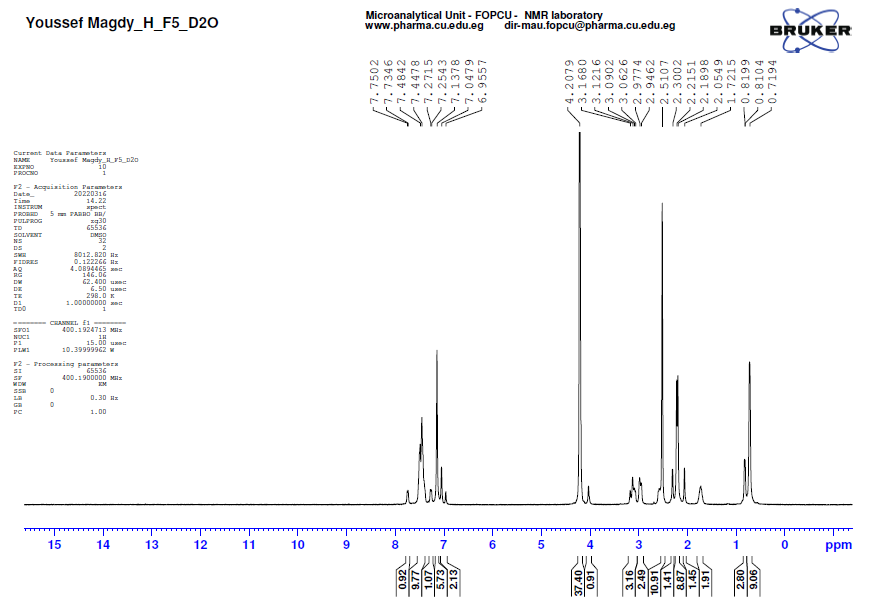

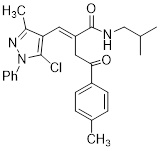

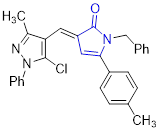

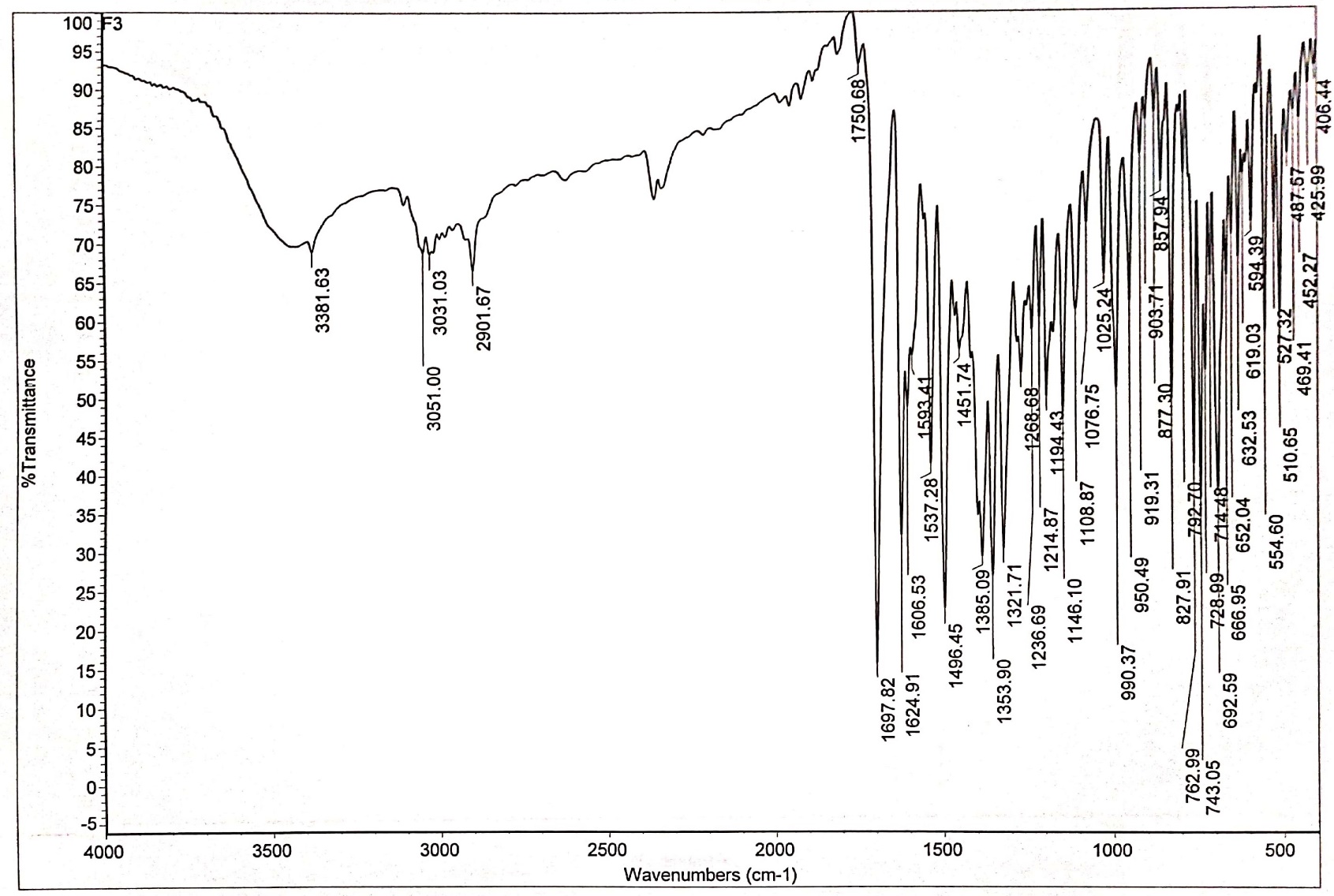

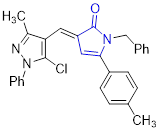

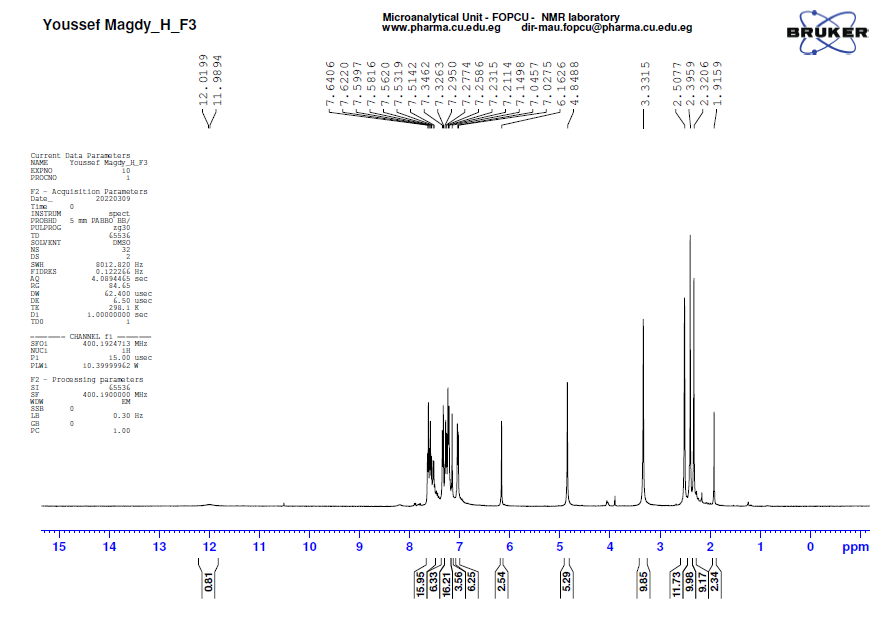

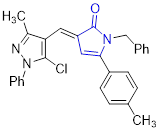

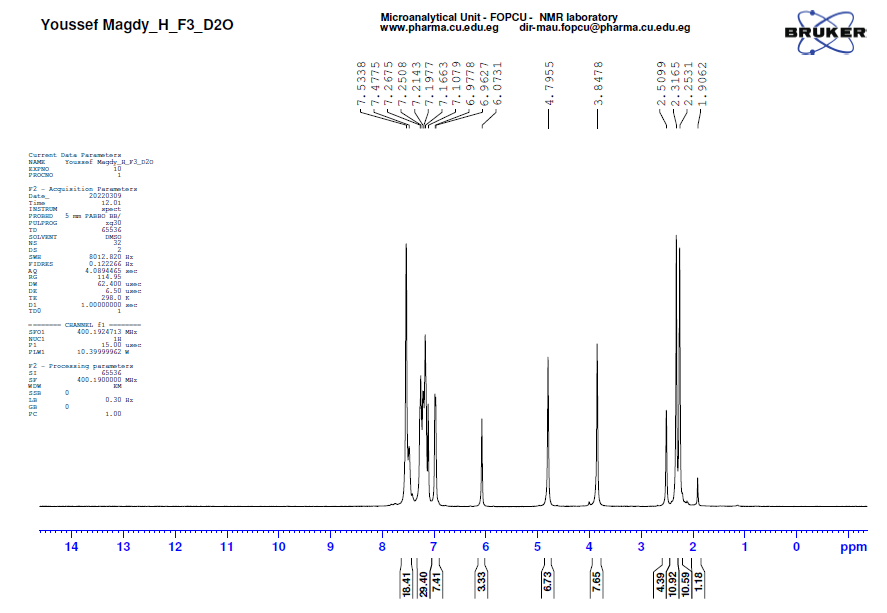

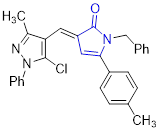

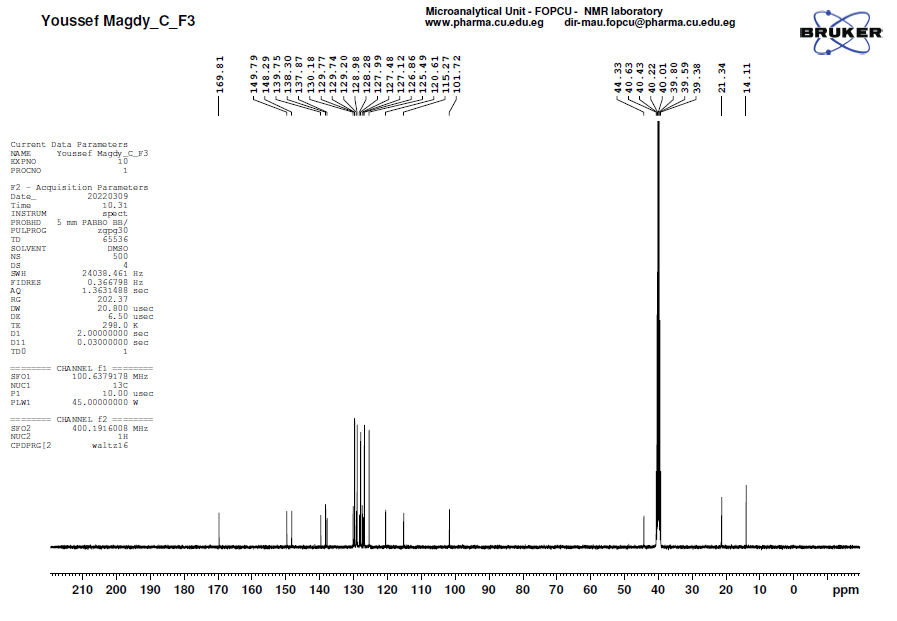

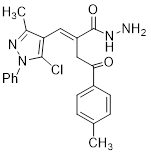

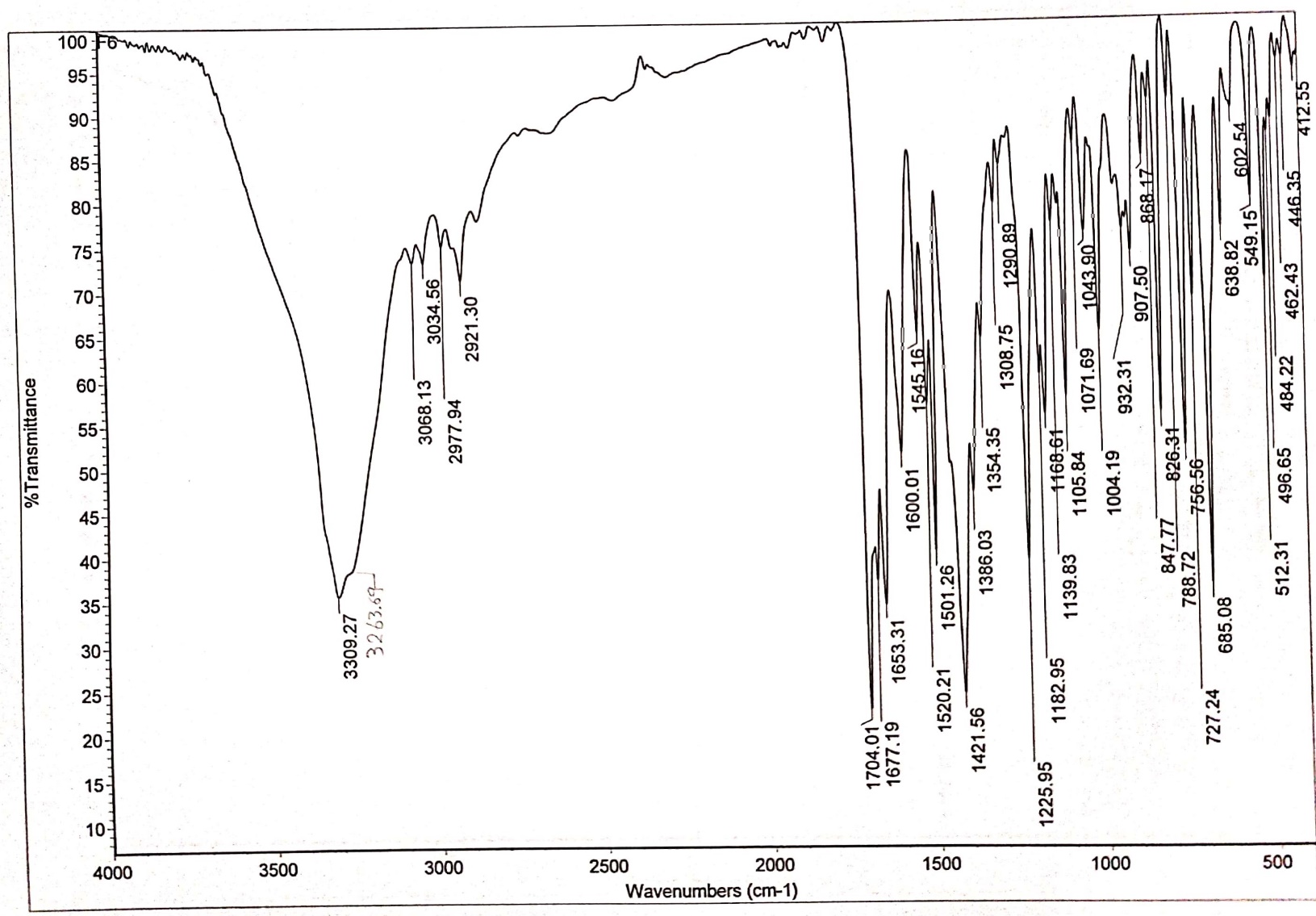

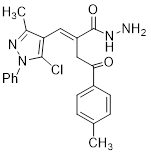

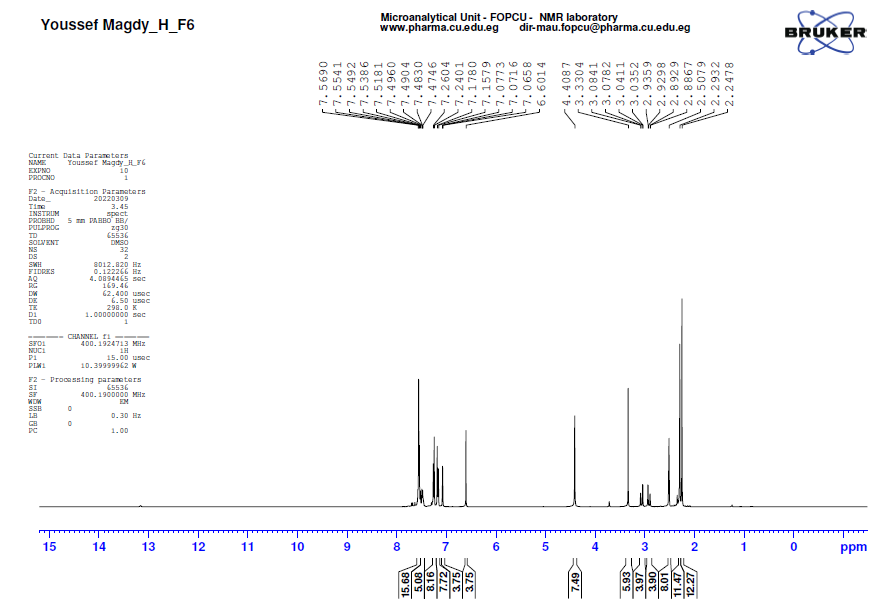

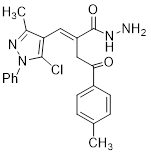

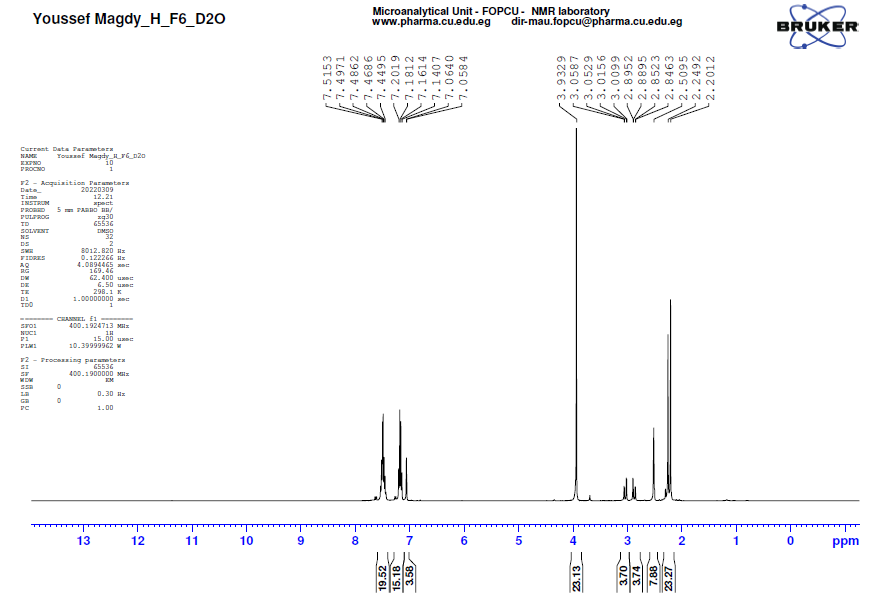

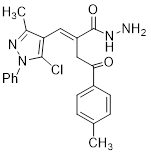

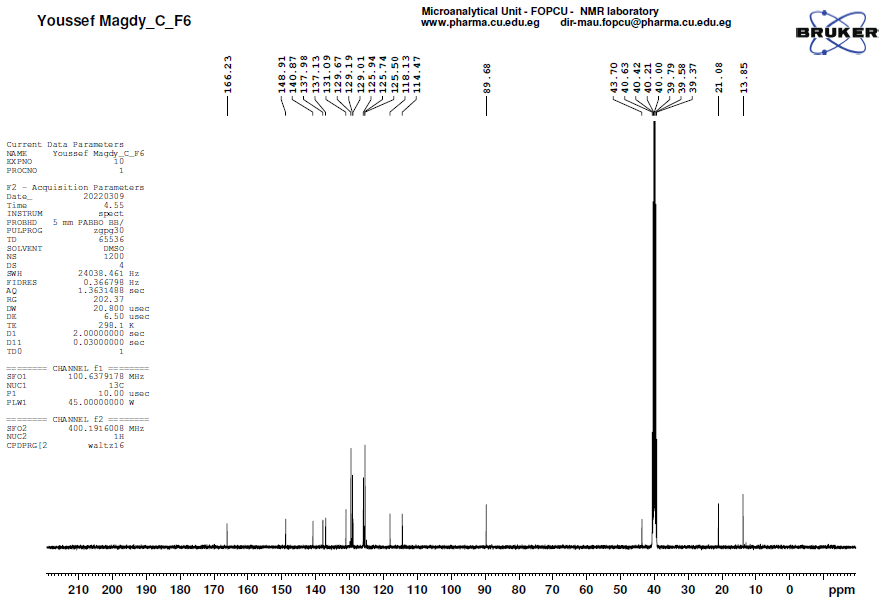

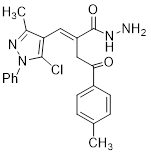

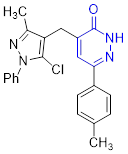

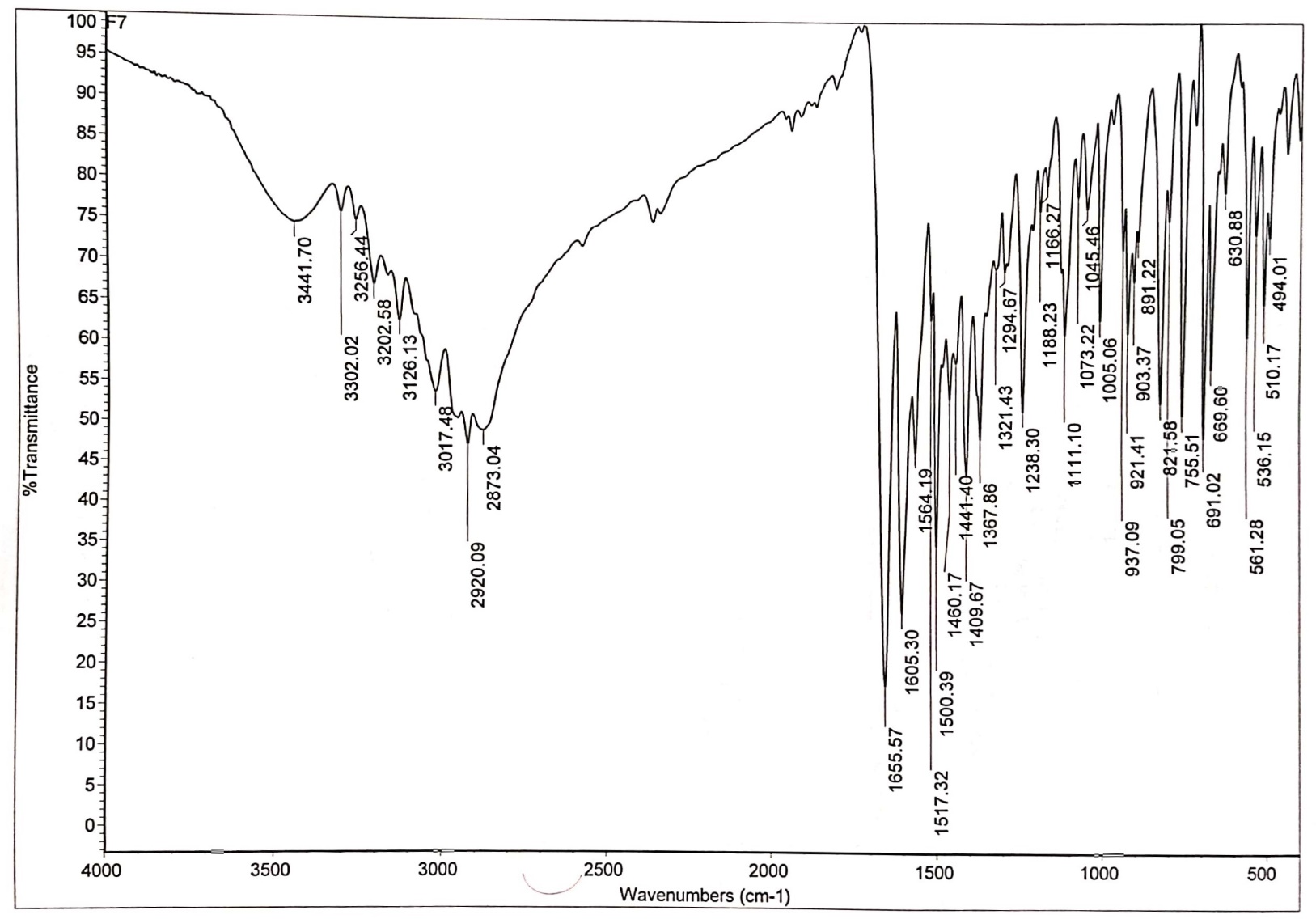

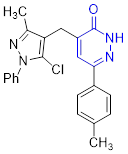

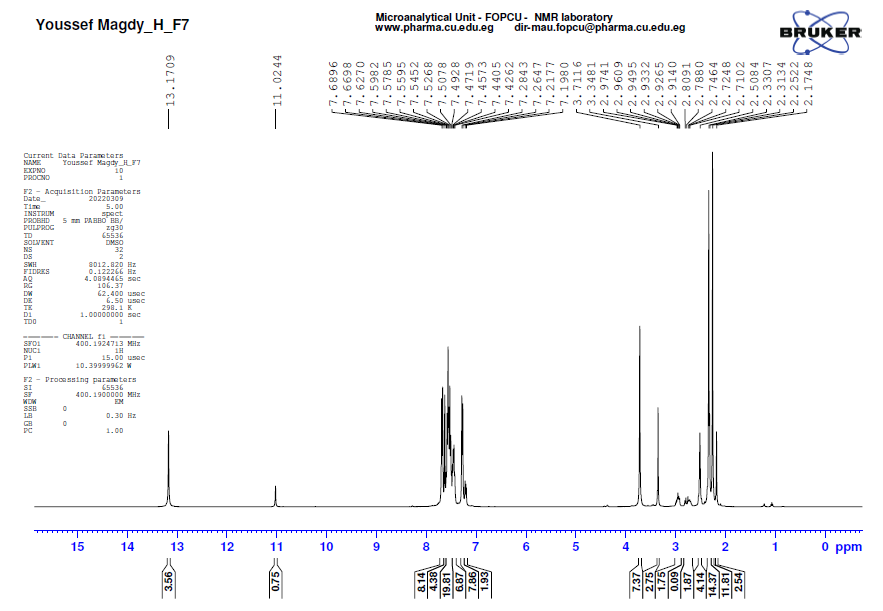

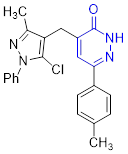

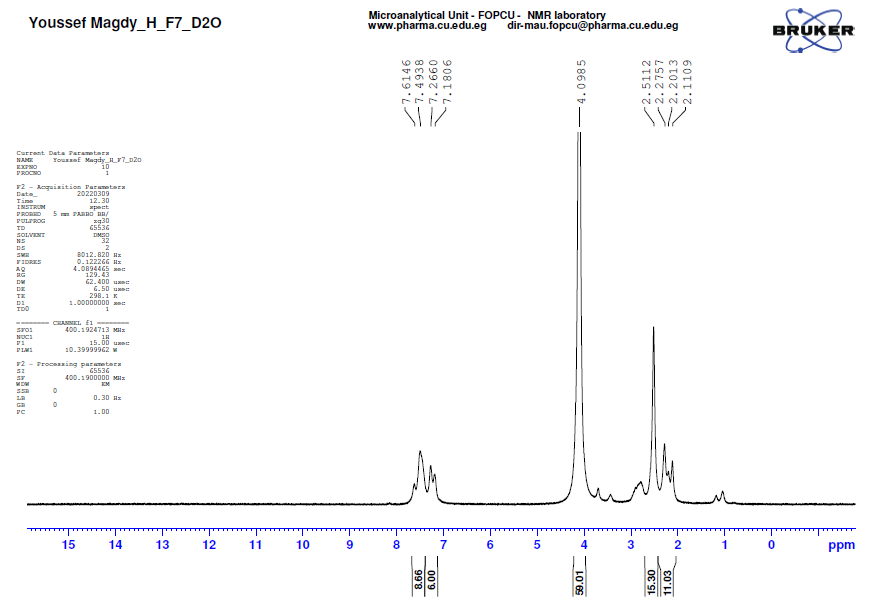

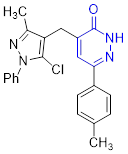

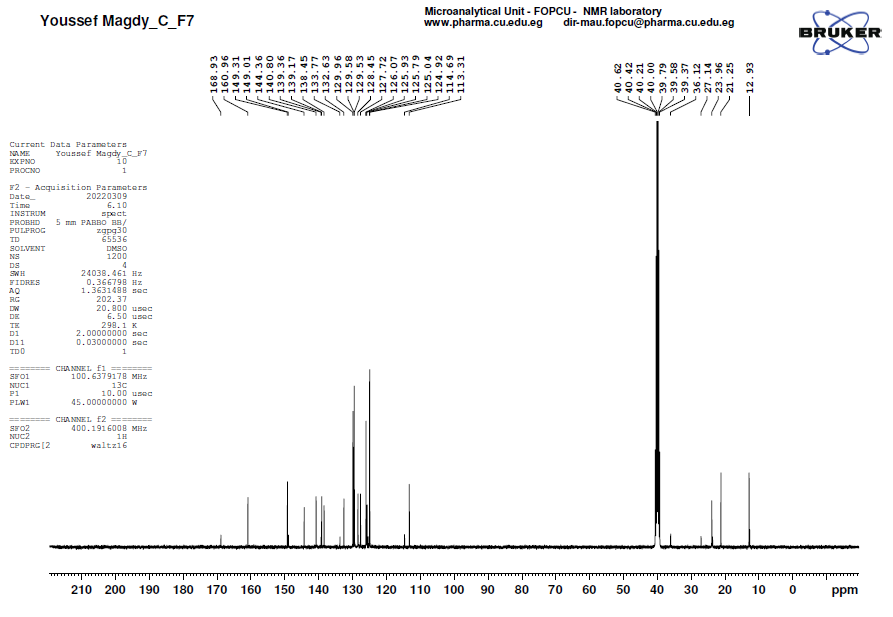

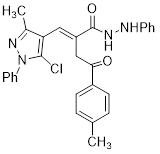

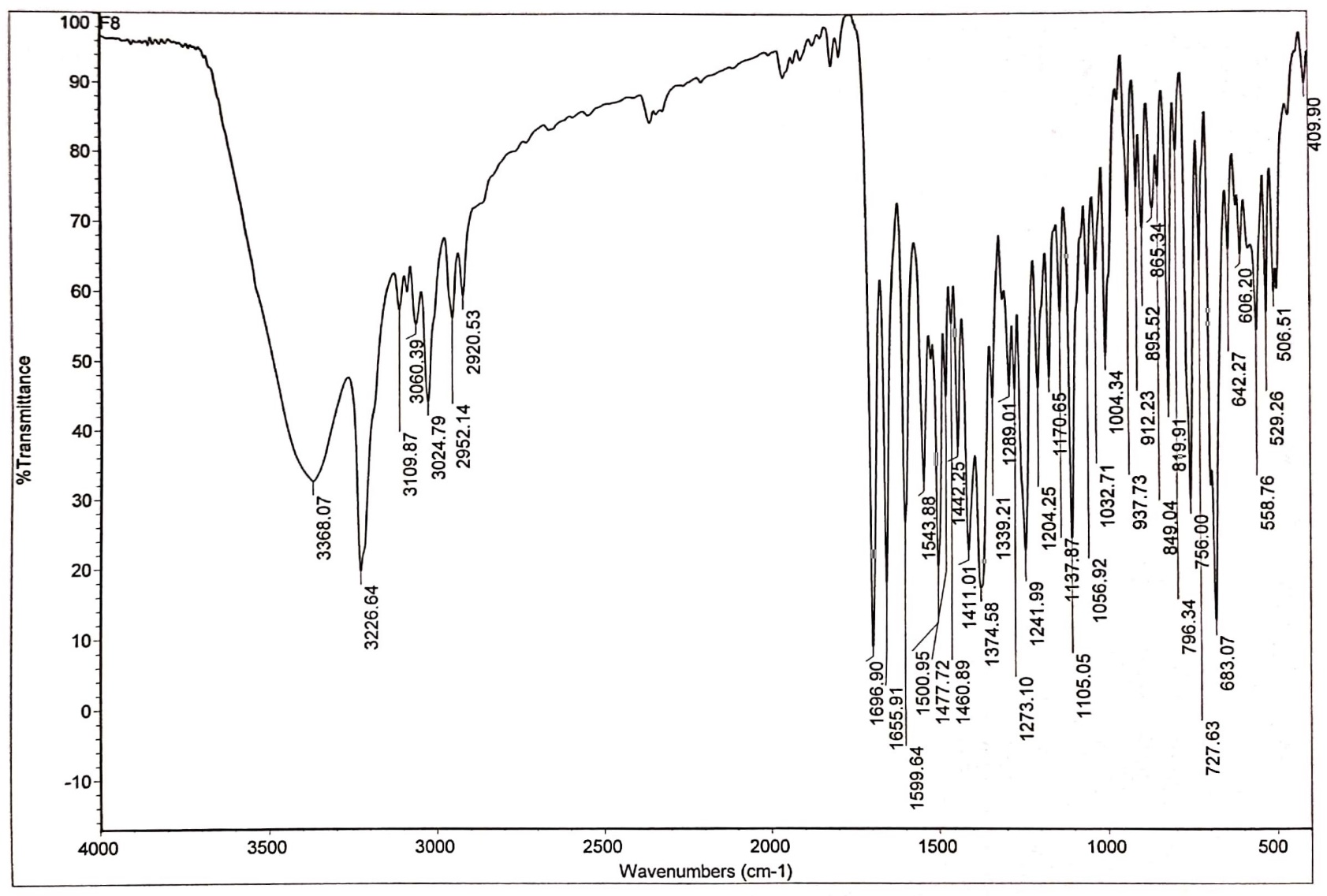

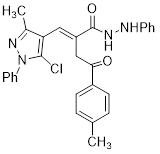

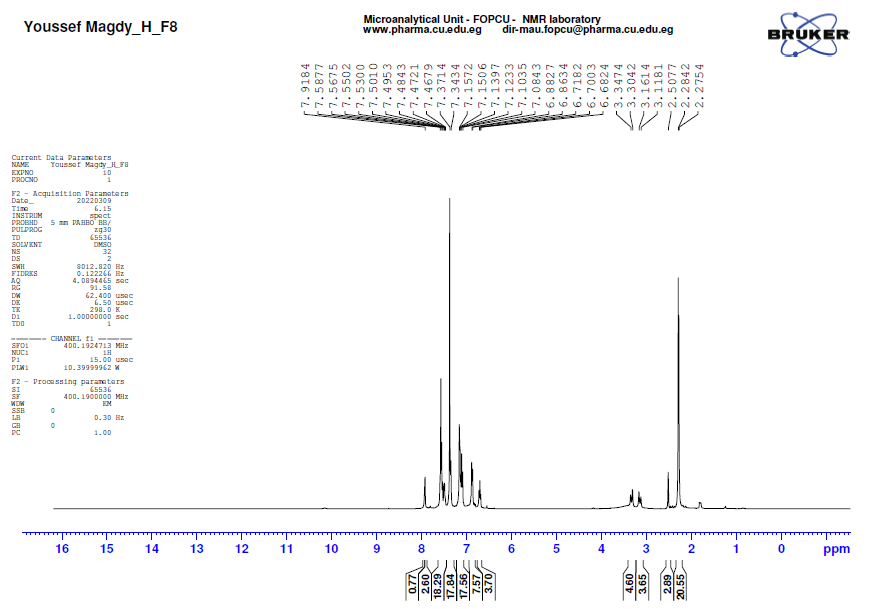

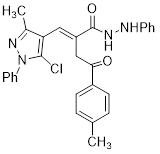

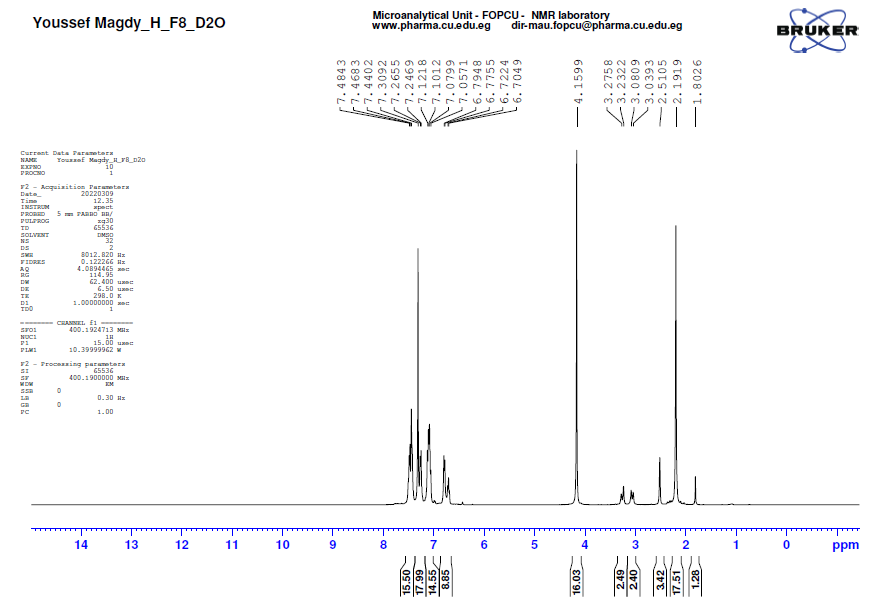

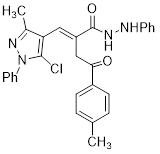

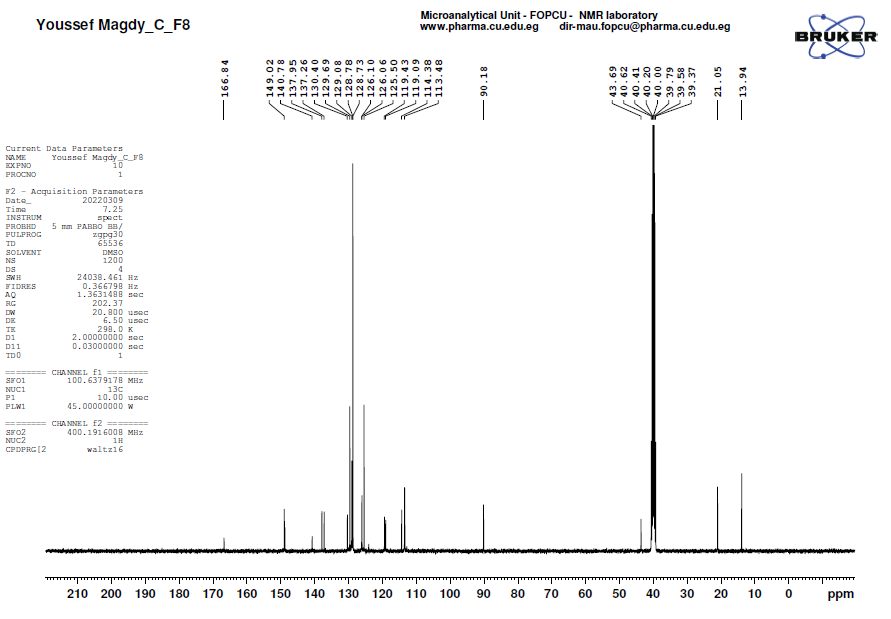

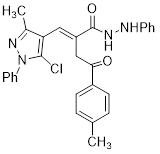

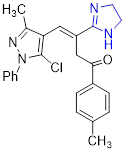

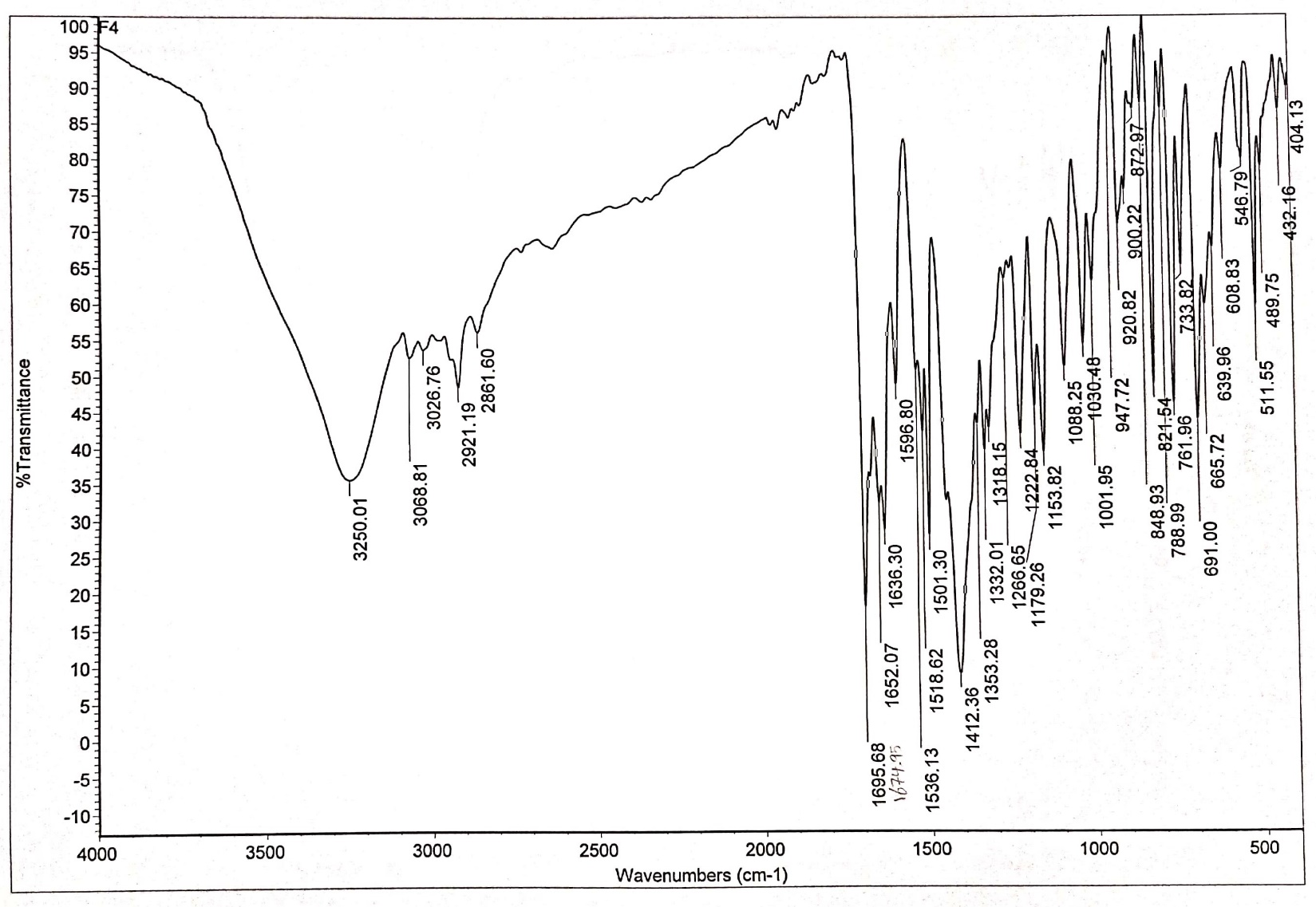

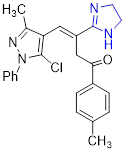

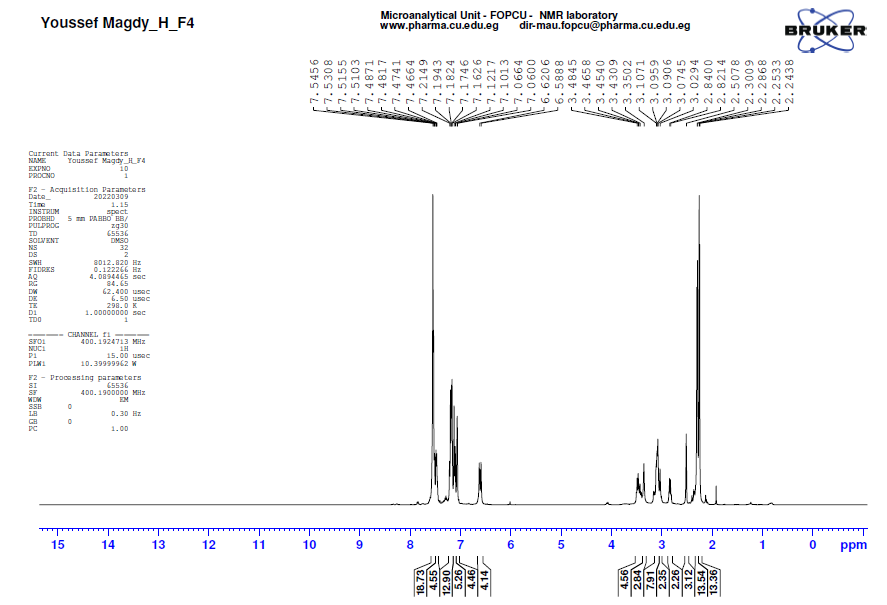

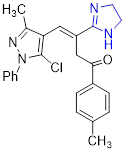

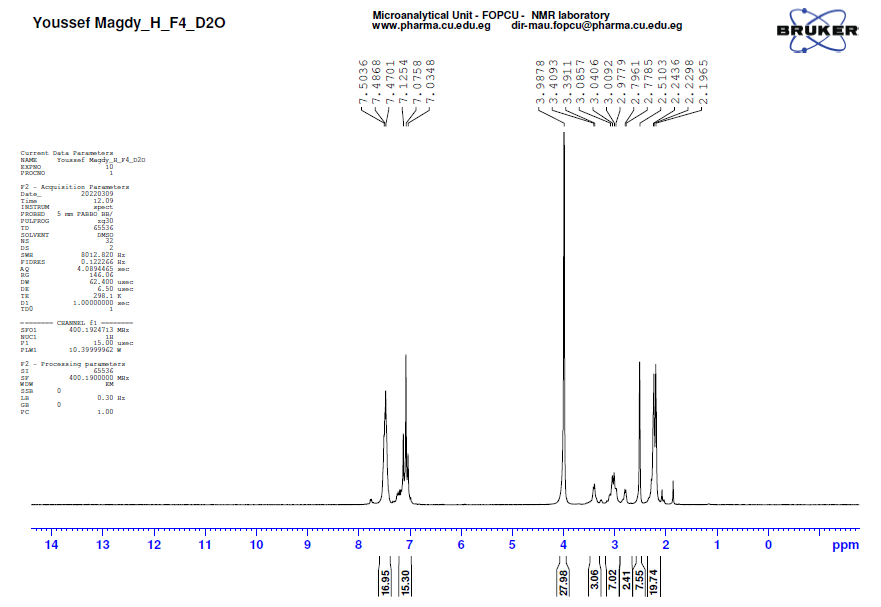

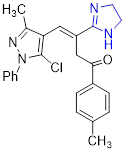

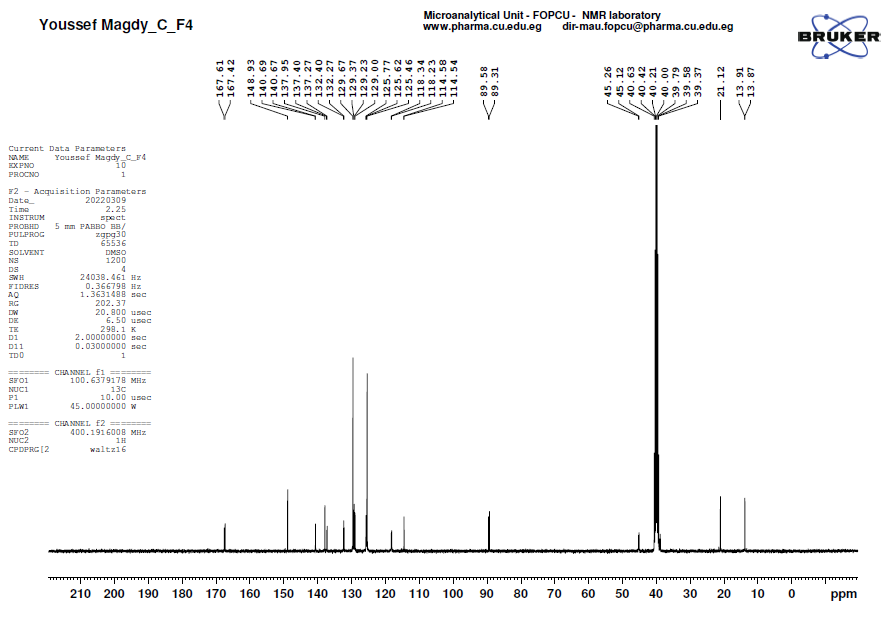

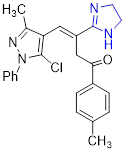

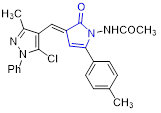

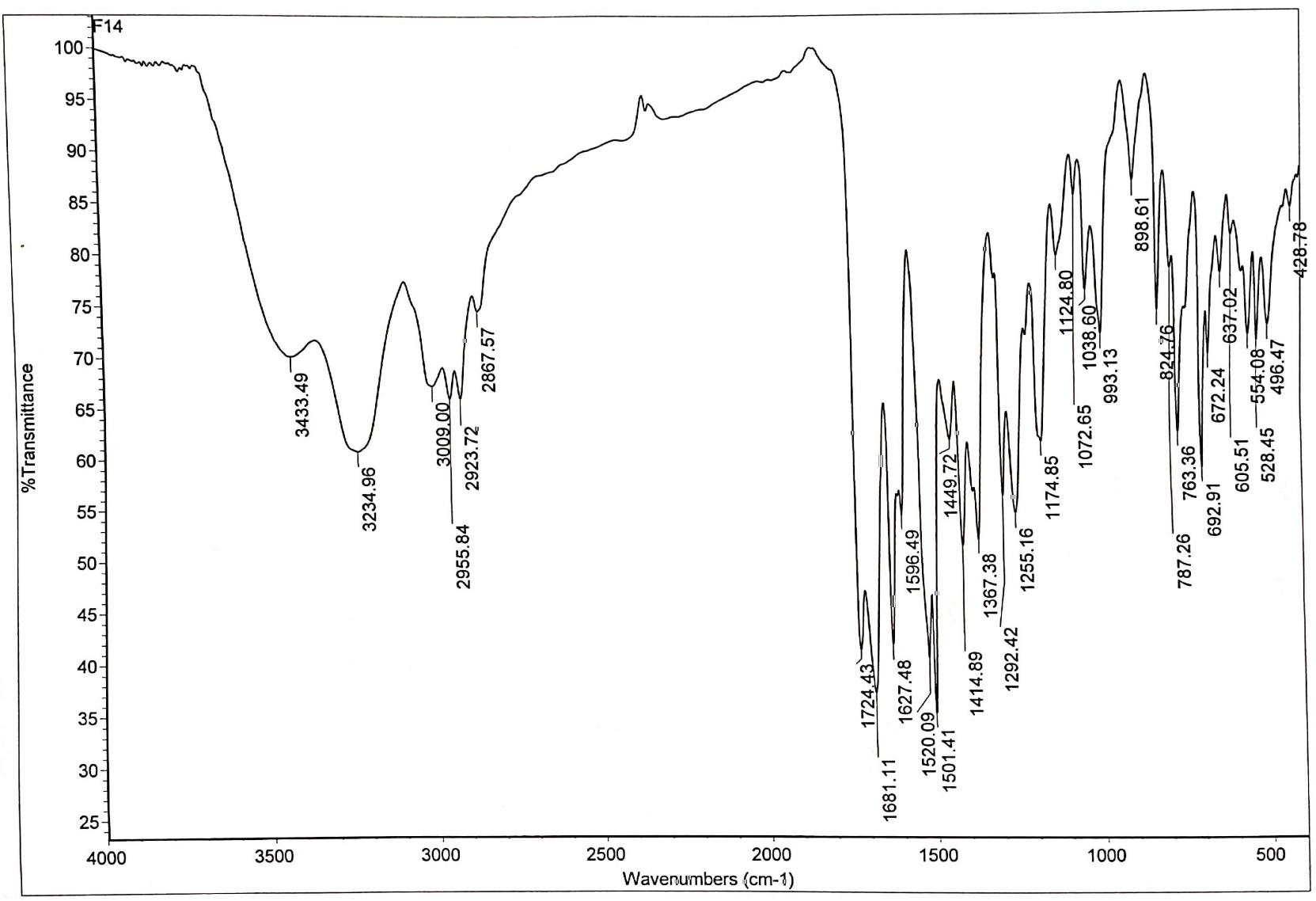

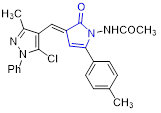

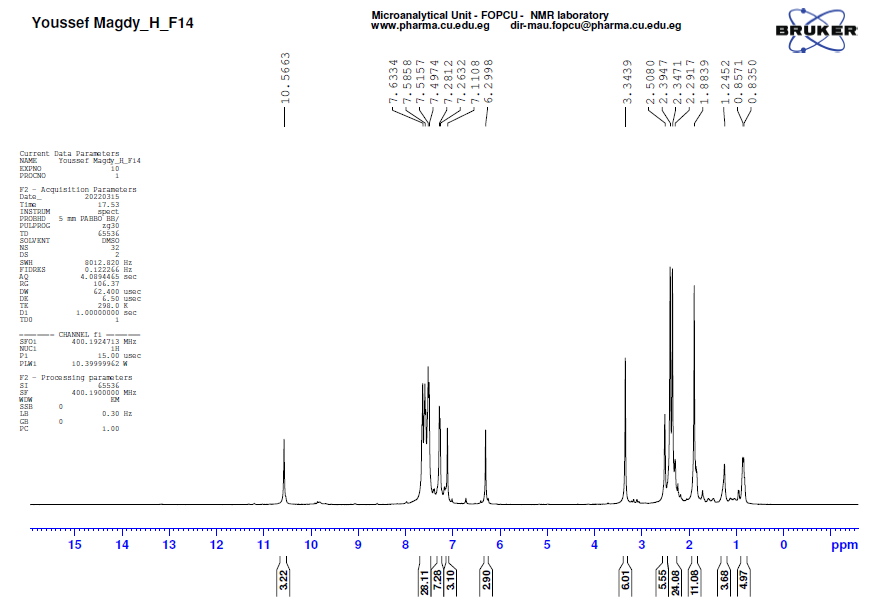

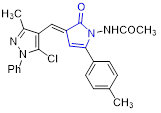

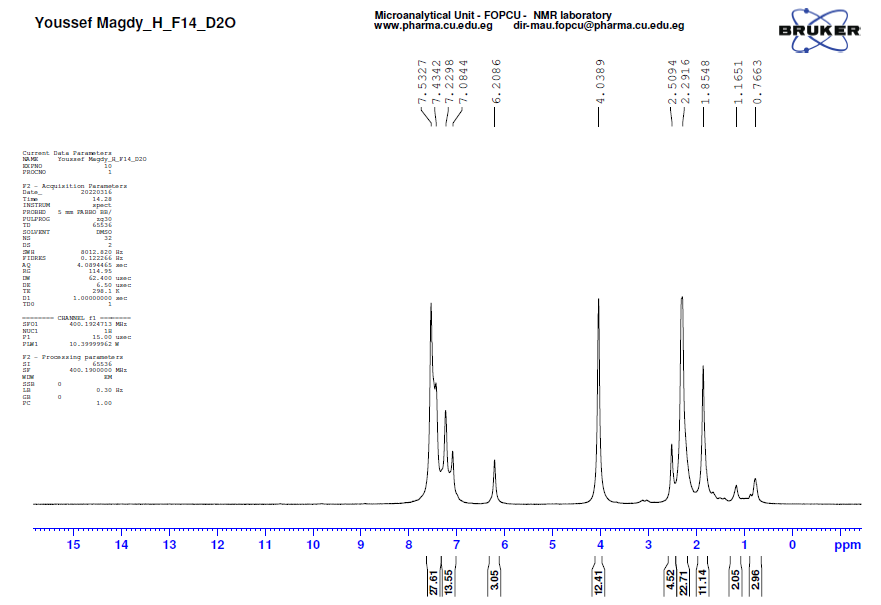

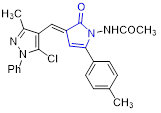

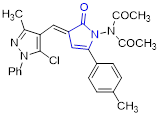

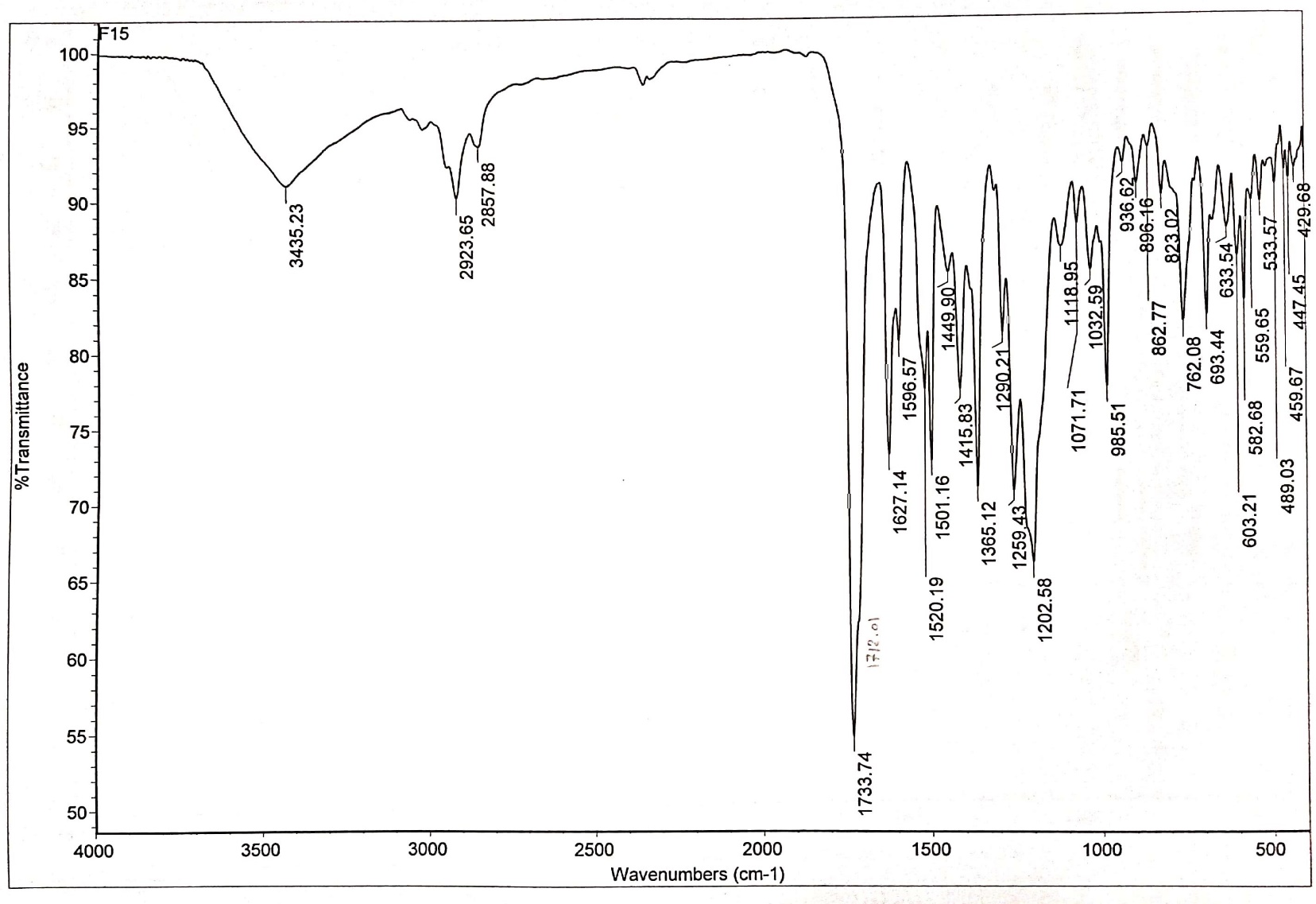

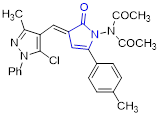

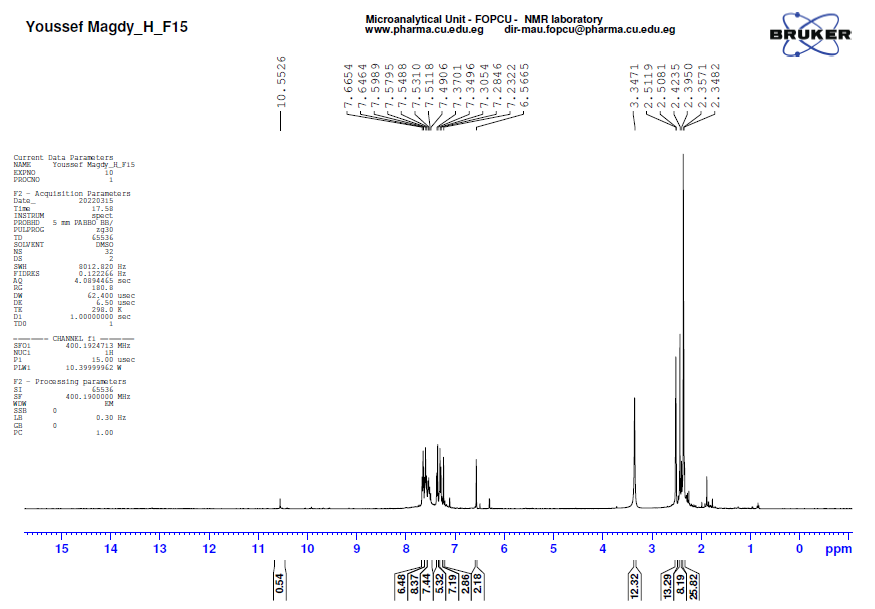

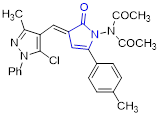

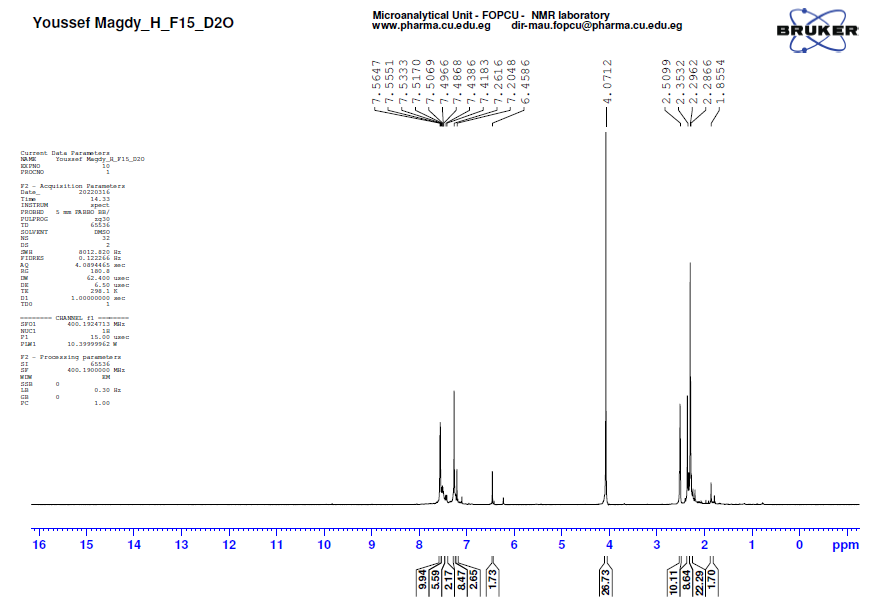

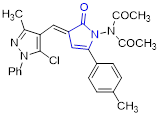

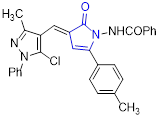

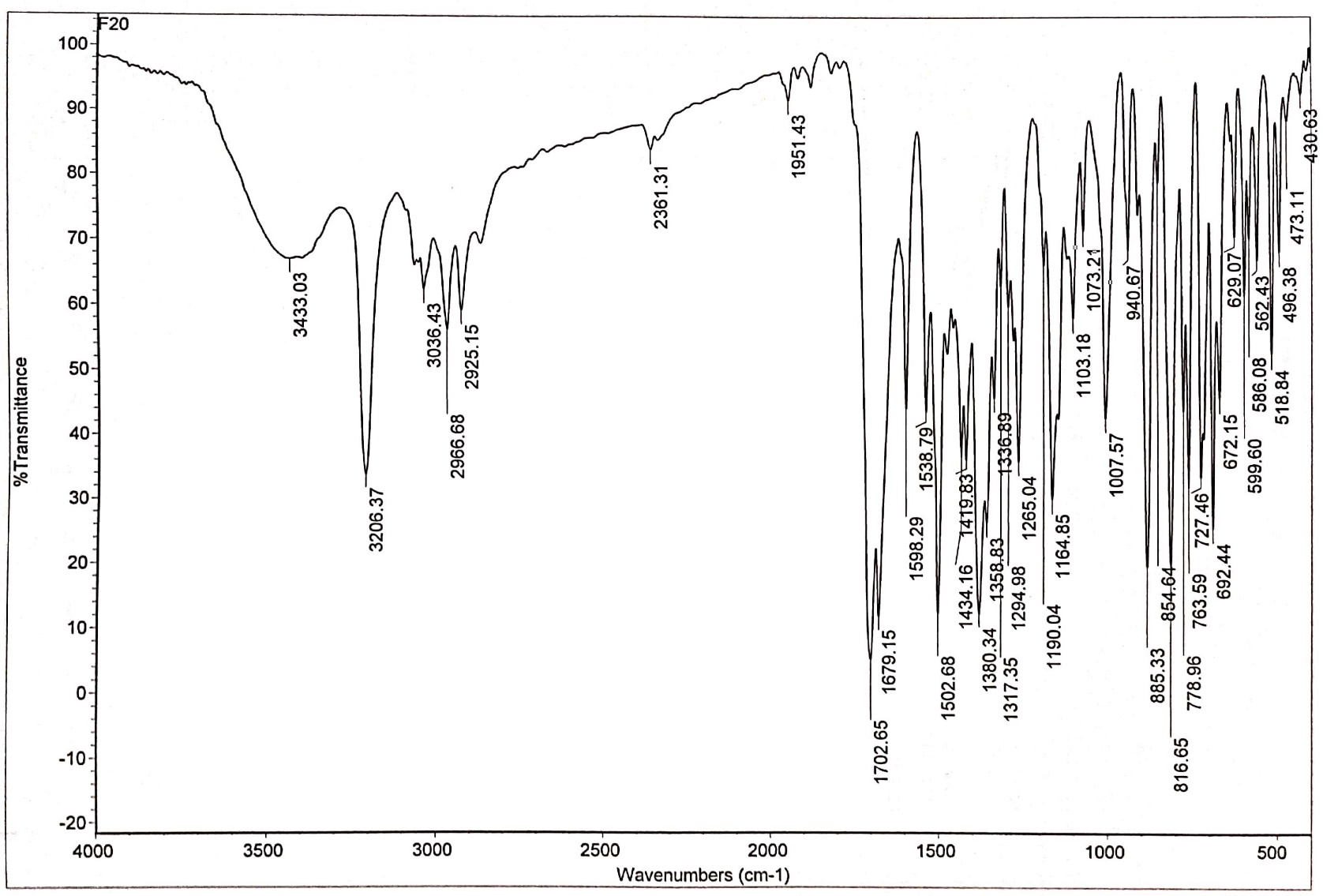

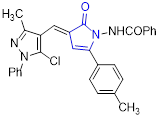

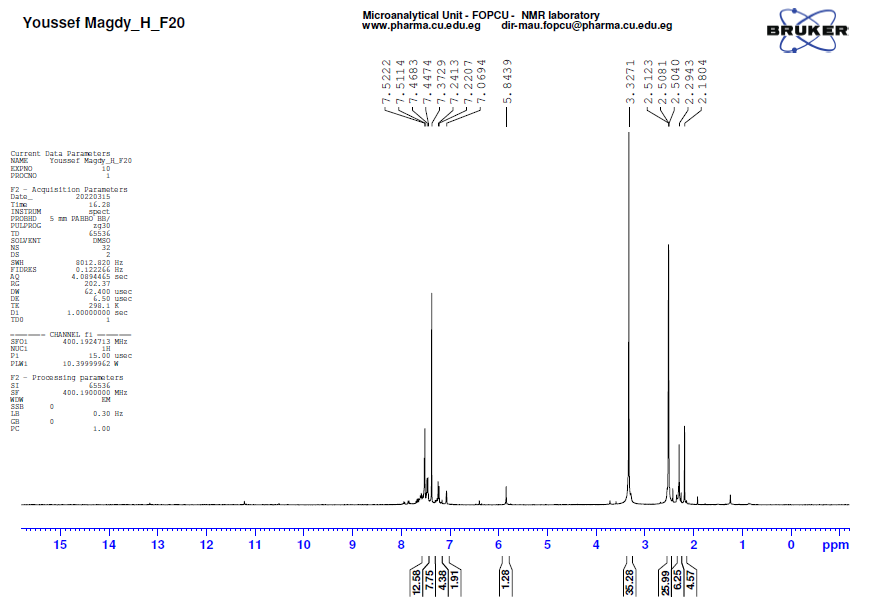

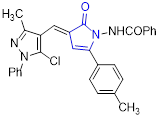

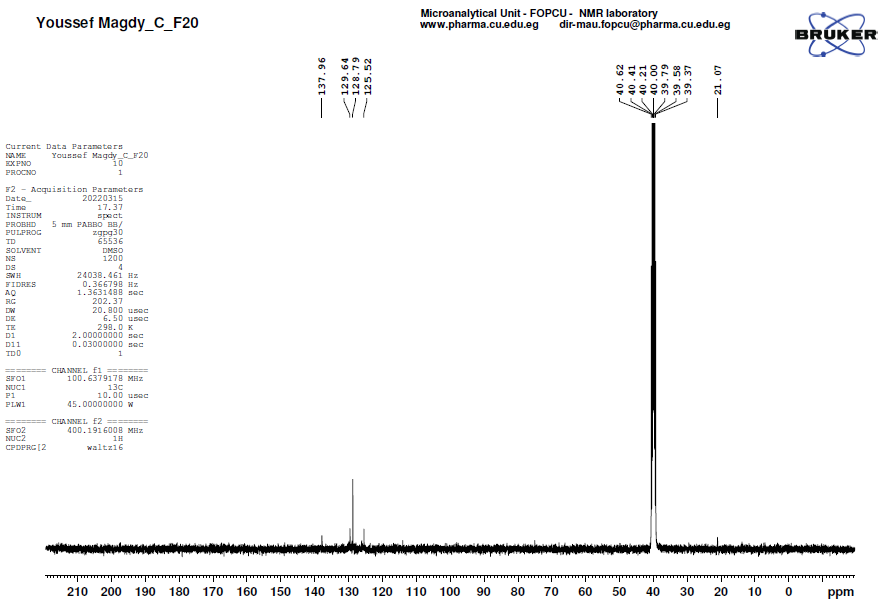

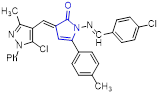

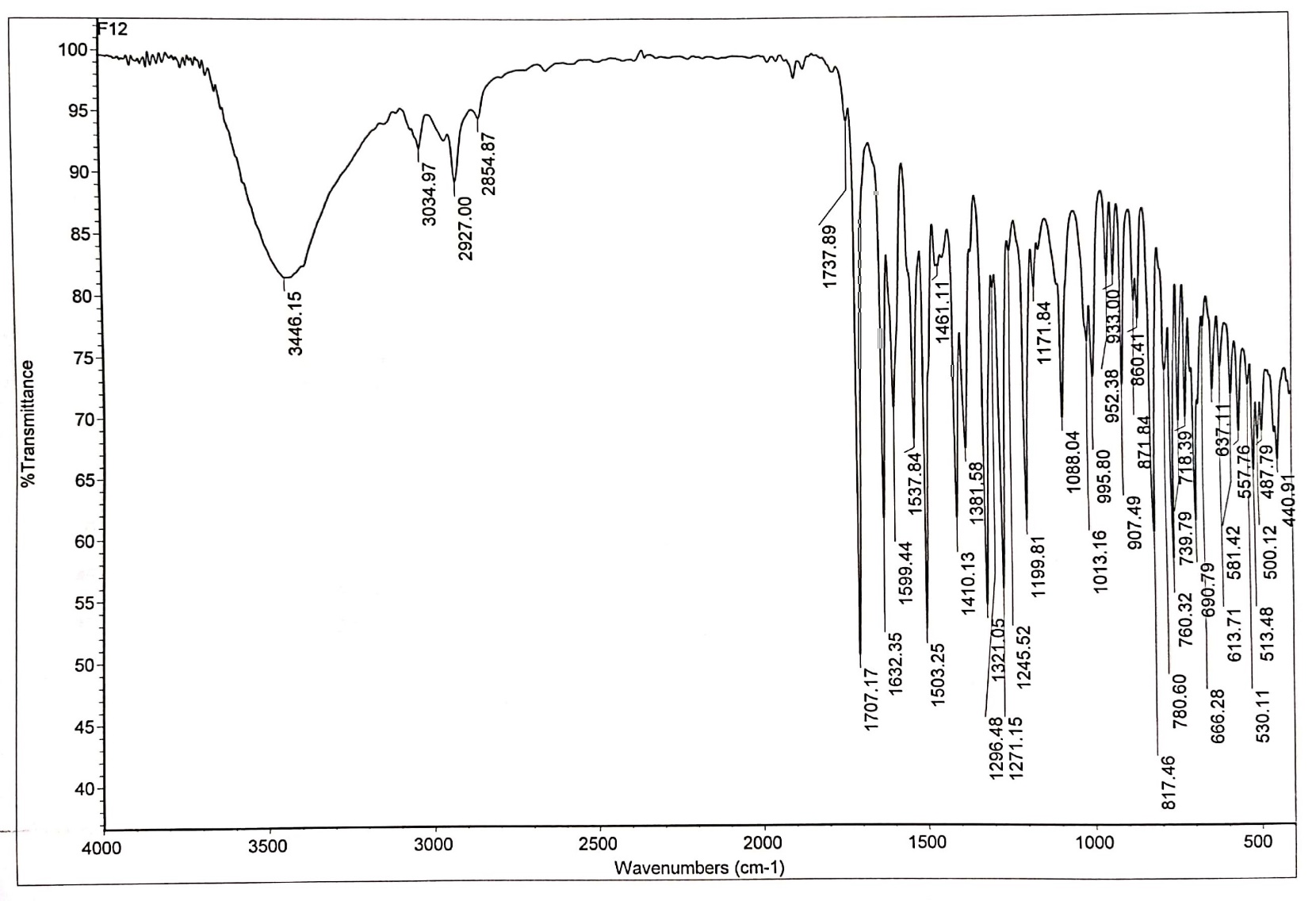

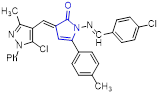

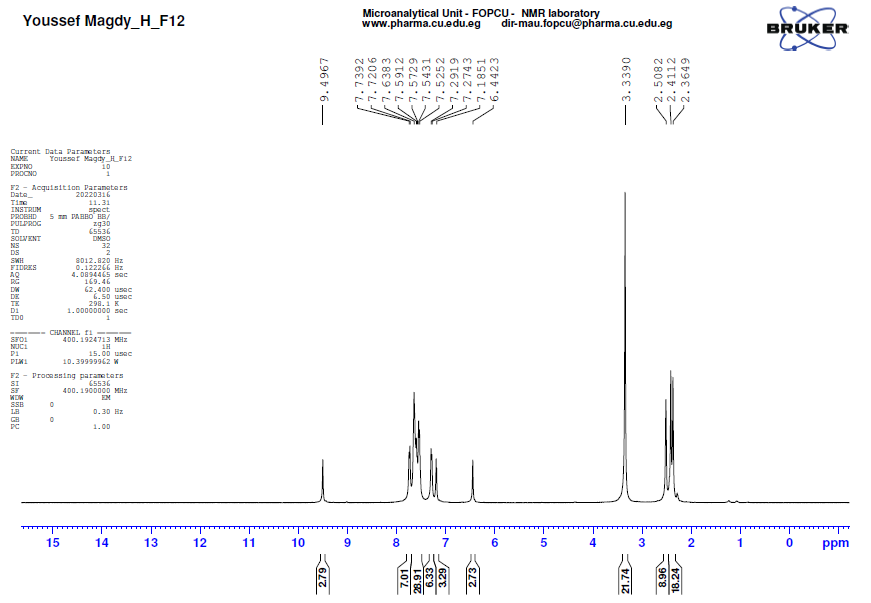


**Fig. 7: ^1^H NMR spectrum of compound (4)**

**Fig. 8: ^1^H NMR-D_2_O spectrum of compound (4)**

**Fig. 9: ^13^C NMR spectrum of compound (4)**

**Fig. 10: Mass spectrum of compound (4)**

**Fig. 11: IR spectrum of compound (5)**

**Fig. 12: ^1^H NMR spectrum of compound (5)**

**Fig. 13: ^1^H NMR-D_2_O spectrum of compound (5)**

**Fig. 14: Mass spectrum of compound (5)**

**Fig. 15: IR spectrum of compound (6)**

**Fig. 16: ^1^H NMR spectrum of compound (6)**

**Fig. 17: ^1^H NMR-D_2_O spectrum of compound (6)**

**Fig. 18: Mass spectrum of compound (6)**

**Fig. 19: IR spectrum of compound (7)**

**Fig. 20: ^1^H NMR spectrum of compound (7)**

**Fig. 21: ^1^H NMR-D_2_O spectrum of compound (7)**

**Fig. 22: ^13^C NMR spectrum of compound (7)**

**Fig. 23: IR spectrum of compound (7)**

**Fig. 24: ^1^HNMR spectrum of compound (7)**

**Fig. 25: ^1^HNMR-D_2_O spectrum of compound (7)**

**Fig. 26: ^13^CNMR spectrum of compound (7)**

**Fig. 23: IR spectrum of compound (8)**

**Fig. 24: ^1^H NMR spectrum of compound (8)**

**Fig. 25: ^1^H NMR-D_2_O spectrum of compound (8)**

**Fig. 26: ^13^CNMR spectrum of compound (8)**

**Fig. 27: Mass spectrum of compound (8)**

**Fig. 28: IR spectrum of compound (9)**

**Fig. 29: ^1^H NMR spectrum of compound (9)**

**Fig. 30: ^1^HNMR-D_2_O spectrum of compound (9)**

**Fig. 31: ^13^CNMR spectrum of compound (9)**

**Fig. 32: IR spectrum of compound (10)**

**Fig. 33: ^1^HNMR spectrum of compound (10)**

**Fig. 34: ^1^HNMR-D_2_O spectrum of compound (10)**

**Fig. 35: ^13^CNMR spectrum of compound (10)**

**Fig. 36: Mass spectrum of compound (10)**

**Fig. 37: IR spectrum of compound (11)**

**Fig. 38: ^1^HNMR spectrum of compound (11)**

**Fig. 39: ^1^HNMR-D_2_O spectrum of compound (11)**

**Fig. 40: ^13^CNMR spectrum of compound (11)**

**Fig. 41: Mass spectrum of compound (11)**

**Fig. 42: IR spectrum of compound (12)**

**Fig. 43: ^1^H NMR spectrum of compound (12)**

**Fig. 44: ^1^HNMR-D_2_O spectrum of compound (12)**

**Fig. 45: Mass spectrum of compound (12)**

**Fig. 46: IR spectrum of compound (13)**

**Fig. 47: ^1^H NMR spectrum of compound (13)**

**Fig. 48: ^1^H NMR-D_2_O spectrum of compound (13)**

**Fig. 49: Mass spectrum of compound (13)**

**Fig. 50: IR spectrum of compound (14)**

**Fig. 51: ^1^HNMR spectrum of compound (14)**

**Fig. 52: ^13^CNMR spectrum of compound (14)**

**Fig. 53: IR spectrum of compound (15)**

**Fig. 54: ^1^H NMR spectrum of compound (15)**

**Fig. 55: ^13^C NMR spectrum of compound (15)**

**Fig. 55: Mass spectrum of compound (15)**

**Fig. 56: IR spectrum of compound (16)**

**Fig. 57: ^1^H NMR spectrum of compound (16)**

**Fig. 61: ^1^H NMR-D_2_O spectrum of compound (16)**

**Fig. 58: ^13^C NMR spectrum of compound (16)**

**Fig. 59: Mass spectrum of compound (16)**
